# Structure and allosteric regulation of human NAD-dependent isocitrate dehydrogenase

**DOI:** 10.1038/s41421-020-00220-7

**Published:** 2020-12-22

**Authors:** Pengkai Sun, Yan Liu, Tengfei Ma, Jianping Ding

**Affiliations:** 1 State Key Laboratory of Molecular Biology, Shanghai Institute of Biochemistry and Cell Biology, Center for Excellence in Molecular Cell Science, University of Chinese Academy of Sciences, Chinese Academy of Sciences, 320 Yueyang Road, Shanghai 200031, China; 2 School of Life Science and Technology, ShanghaiTech University, 393 Huaxia Zhong Road, Shanghai 201210, China; 3 School of Life Science, Hangzhou Institute for Advanced Study, University of Chinese Academy of Sciences, 1 Xiangshan Road, Hangzhou, Zhejiang 310024 China

**Keywords:** X-ray crystallography, Protein folding

## Abstract

Human NAD-dependent isocitrate dehydrogenase or HsIDH3 catalyzes the decarboxylation of isocitrate into α-ketoglutarate in the TCA cycle. HsIDH3 exists and functions as a heterooctamer composed of the αβ and αγ heterodimers, and is regulated allosterically and/or competitively by numerous metabolites including CIT, ADP, ATP, and NADH. In this work, we report the crystal structure of HsIDH3 containing a β mutant in apo form. In the HsIDH3 structure, the αβ and αγ heterodimers form the α_2_βγ heterotetramer via their clasp domains, and two α_2_βγ heterotetramers form the (α_2_βγ)_2_ heterooctamer through insertion of the N-terminus of the γ subunit of one heterotetramer into the back cleft of the β subunit of the other heterotetramer. The functional roles of the key residues at the allosteric site, the pseudo allosteric site, the heterodimer and heterodimer–heterodimer interfaces, and the N-terminal of the γ subunit are validated by mutagenesis and kinetic studies. Our structural and biochemical data together demonstrate that the allosteric site plays an important role but the pseudo allosteric site plays no role in the allosteric activation of the enzyme; the activation signal from the allosteric site is transmitted to the active sites of both αβ and αγ heterodimers via the clasp domains; and the N-terminal of the γ subunit plays a critical role in the formation of the heterooctamer to ensure the optimal activity of the enzyme. These findings reveal the molecular mechanism of the assembly and allosteric regulation of HsIDH3.

## Introduction

In all aerobic organisms, the cells use the tricarboxylic acid (TCA) cycle (also called citric acid cycle or Krebs cycle) to generate ATP through oxidation of acetyl-CoA derived from carbohydrates, fats, and proteins. In addition, the TCA cycle also provides intermediates for de novo synthesis of proteins, lipids and nucleic acids^1^. Among a series of biochemical reactions in the TCA cycle, isocitrate dehydrogenases (IDHs) catalyze oxidative decarboxylation of isocitrate (ICT) into α-ketoglutarate (α-KG) using NAD or NADP as coenzyme. Most prokaryotes contain only NADP-dependent IDHs (NADP-IDHs) in the cytosol to exert the catalytic function. Eukaryotes contain both NADP-IDHs and NAD-dependent IDHs (NAD-IDHs). In human and other mammalian cells, there are two NADP-IDHs, which are located to the cytosol and the mitochondria, and one NAD-IDH which is located to the mitochondria. Human (*Homo sapien*) NADP-IDHs and NAD-IDH are also called HsIDH1, HsIDH2, and HsIDH3, respectively. It is well established that HsIDH3 exerts the catalytic function in the TCA cycle^2^, whereas HsIDH1 and HsIDH2 play important roles in cellular defense against oxidative damage^3^, removal of reactive oxygen species^4^, and synthesis of fat and cholesterol^5^. Aberrant functions of all three enzymes have been implicated in the pathogenesis of numerous metabolic diseases^6–8^ and malignant tumors^9–12^.

Both prokaryotic^13^ and eukaryotic^14,15^ NADP-IDHs exist and function as homodimers in which both subunits have catalytic activity. These enzymes share a conserved catalytic mechanism, but have different regulatory mechanisms. The activity of *Escherichia coli* NADP-IDH is regulated through reversible phosphorylation and dephosphorylation of a strictly conserved Ser at the active site by the dual functional kinase/phosphatase AceK, and other bacterial NADP-IDHs might share a similar regulatory mechanism^16,17^. The activity of HsIDH1 is regulated through substrate-binding induced conformational change of a key structure element at the active site, and other mammalian NADP-IDHs might utilize a similar regulatory mechanism^14,18^.

Compared to NADP-IDHs, NAD-IDHs are composed of different types of subunits with distinct functions and employ more sophisticated regulatory mechanisms. *Saccharomyces cerevisiae* NAD-IDH is composed of a regulatory subunit ScIDH1 and a catalytic subunit ScIDH2, which form the ScIDH1/ScIDH2 heterodimer as the basic functional unit^19^, and the heterodimer is assembled into a heterotetramer and further into a heterooctamer^20,21^. ScIDH2 contains the active site and ScIDH1 contains the allosteric site, and the binding of activators citrate (CIT) and AMP to the allosteric site causes conformational changes of the active site through the heterodimer interface, leading to activation of the enzyme^20^. The composition and regulation of mammalian NAD-IDHs are more complex than those of yeast NAD-IDH. Mammalian NAD-IDH is composed of three types of subunits in the ratio of 2αː1βː1γ ^22,23^. The α, β, and γ subunits have a molecular mass of about 37, 39, and 39 kDa, respectively; and the α and β subunits share about 40% sequence identity, the α and γ subunits about 42% sequence identity, and the β and γ subunits about 52% sequence identity. The α and β subunits form a heterodimer (αβ) and the α and γ subunits form another heterodimer (αγ), and the two heterodimers are assembled into the α_2_βγ heterotetramer and further into the (α_2_βγ)_2_ heterooctamer^24^. Early biochemical studies of mammalian NAD-IDHs showed that the α subunit is the catalytic subunit^25^, and the β and γ subunits are the regulatory subunits^26^; and the activity is positively regulated by CIT^27^ and ADP^28^, but negatively regulated by ATP and NADH^29^. The functional roles of several strictly conserved residues of the α, β, and γ subunits of HsIDH3 in the bindings of metal ion, ICT, NAD, and ADP and the catalytic reaction were also examined by biochemical studies, and the results showed that the α subunit is critical for the catalytic activity, and the β and γ subunits play important roles in the allosteric regulation^30–34^.

Our biochemical and structural studies of HsIDH3 confirmed some results from the previous studies but also revealed some new findings. We found that the α subunits of both αβ and αγ heterodimers have catalytic activity; however, only the γ subunit plays a regulatory role via an allosteric regulatory mechanism, while the β subunit plays no regulatory role but is required for the optimal function of HsIDH3^35^. The αγ heterodimer are positively regulated by CIT and ADP^36^, and negatively regulated by NADH^37^. In addition, these enzymes can be activated by low concentrations of ATP but inhibited by high concentrations of ATP^38^. In contrast, the αβ heterodimer cannot be activated by CIT and ADP and is inhibited by both NADH and ATP^39^. Our findings revealed the underlying molecular mechanisms of the αγ and αβ heterodimers. Nevertheless, so far the structure, assembly and regulatory mechanism of HsIDH3 remain unknown. Thus, how the αβ and αγ heterodimers are assembled into the α_2_βγ heterotetramer and further into the (α_2_βγ)_2_ heterooctamer is unclear. How the allosteric site in the γ subunit regulates both α subunits in the α_2_βγ heterotetramer is also unclear. Whether the regulatory mechanisms of the αβ and αγ heterodimers are applicable to the heterooctamer is elusive.

In this work, we determined the crystal structure of HsIDH3 containing a β mutant in apo form. In the apo HsIDH3 structure, the αβ and αγ heterodimers assemble the α_2_βγ heterotetramer via their clasp domains, and two α_2_βγ heterotetramers assemble the (α_2_βγ)_2_ heterooctamer through insertion of the N-terminal of the γ subunit of one heterotetramer into the back cleft of the β subunit of the other heterotetramer. We also performed mutagenesis and kinetic studies to validate the functional roles of key residues at the allosteric site, the pseudo allosteric site, the heterodimer interface, and the heterodimer–heterodimer interface, as well as the N-terminal of the γ subunit. Our structural and biochemical data together reveal the molecular mechanism for the assembly and allosteric regulation of HsIDH3.

## Results

### Preparation and biochemical analysis of HsIDH3

Crystallization of wild-type HsIDH3 yielded crystals which diffracted X-rays to low resolution (about 10 Å), prohibiting us from determining the crystal structure. Our previous biochemical and structural studies showed that substitution of the C-terminal of the β subunit (residues 341–349) with that of the α subunit (residues 330–338) produced a stable αβ mutant which exhibits similar enzymatic properties as wild-type αβ heterodimer, and this αβ mutant yielded high quality crystals which allowed us to solve the structure of the αβ heterodimer^39^. Thus, we prepared a mutant HsIDH3 containing this β mutant, which led to successful structure determination of HsIDH3.

Like wild-type HsIDH3, the β-mutant HsIDH3 exists as a stable heterooctamer in solution with high purity and homogeneity as shown by SEC, SDS-PAGE (Supplementary Fig. S1), and SEC-MALS analyses (Supplementary Fig. S2c, d and Table S1). The β-mutant HsIDH3 exhibits almost identical enzymatic properties as wild-type HsIDH3 (Supplementary Fig. S3a, b and Table S2). These results indicate that the substitution of the C-terminal of the β subunit has no notable effects on the biochemical and enzymatic properties of HsIDH3.

### Crystal structure of HsIDH3 in apo form

**Fig. 1:**
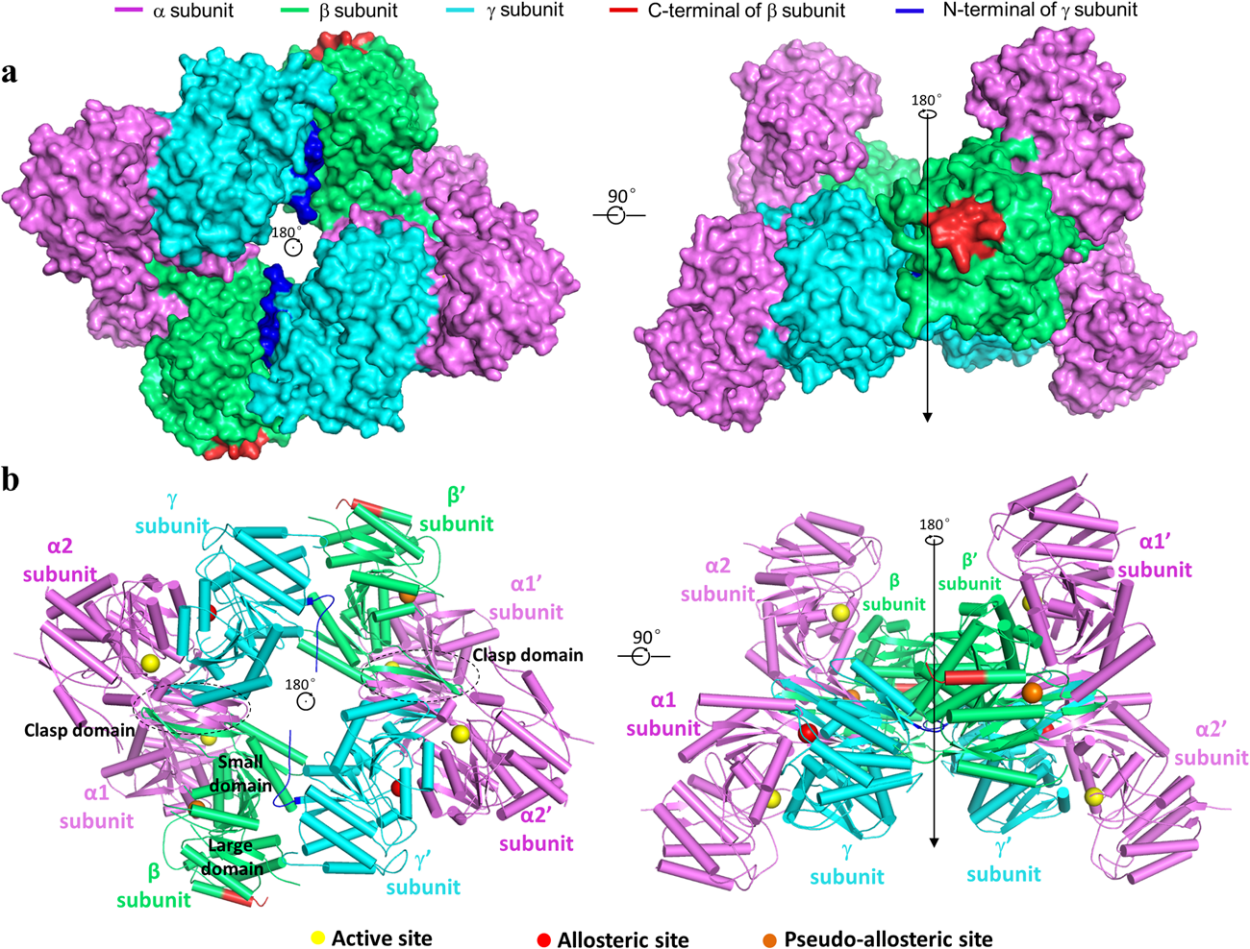
Overall structure of the apo β-mutant HsIDH3. **a** Surface and **b** cartoon presentations of the apo β-mutant HsIDH3 in two different orientations. Left: view along the crystallographic 2-fold axis of the heterooctamer of HsIDH3. Right: view in perpendicular to the crystallographic 2-fold axis of the heterooctamer of HsIDH3. The color coding of the α, β, and γ subunits is shown above. The N-terminal regions of the γ subunits and the C-terminal substituted regions of the β subunits are colored in blue and red, respectively. The clasp domains of the αβ and αγ heterodimers are indicated with dashed ovals. The active sites, the allosteric sites, and the pseudo allosteric sites are indicated with yellow, red, and orange spheres, respectively. Residues and structure elements of the α and β subunits of the αβ heterodimer and the α and γ subunits of the αγ heterodimer are superscripted by “A1” and “B”, and “A2” and “G”, respectively.

The crystal structure of the β-mutant HsIDH3 was solved at 3.47 Å resolution with each asymmetric unit containing one α_2_βγ heterotetramer (Table 1). The four polypeptide chains of the heterotetramer are largely well-defined with good electron density except for a few N-terminal and/or C-terminal residues, and the α, β, and γ subunits can be distinguished unambiguously based on the differences of numerous residues with large side chains (Supplementary Fig. S4). There are no ligands bound at the active sites, the allosteric site, and the pseudo allosteric site; thus, this structure represents the apo HsIDH3. In the apo β-mutant HsIDH3 structure, the αβ and αγ heterodimers assume very similar overall structures as those in the isolated forms^36,39^ (Fig. 1). All of the α, β, and γ subunits consist of 10 α-helices and 12 β-strands, which fold into a large domain, a small domain, and a clasp domain. Both of the αβ and αγ heterodimers have a pseudo two-fold symmetry along the heterodimer interface. The heterodimer interface is mediated by the α6 and α7 helices of the small domains which form a four-helix bundle in a parallel manner, and the β6 and β7 strands of the clasp domains (clasp β-strands), which form a four-stranded β-sheet (clasp β-sheet) in an antiparallel manner. The heterodimer interface buries about 2180 Å^2^ and 2094 Å^2^ solvent accessible surface or 13.7% and 13.5% of the surface area of each subunit in the αβ and αγ heterodimers, respectively, indicating that the heterodimer interface is very tight in both heterodimers.

**Table 1:** Statistics of X-ray diffraction data and structure refinement.

Structure	HsIDH3
Diffraction data	
Wavelength (Å)	0.9792
Space group	*I*4_1_22
Cell parameters	
* a*, *b*, *c* (Å)	204.57, 204.57, 237.88
Resolution (Å)	50.0–3.47 (3.59–3.47)
Observed reflections	585,722
Unique reflections (I/σ (I) > 0)	32,854
Average redundancy	17.8 (17.8)
Average I/σ(I)	35.1 (1.7)
Completeness (%)	100.0 (100.0)
R_merge_ (%)	10.1 (187.5)
CC ½ (%)	99.5 (67.6)
Refinement and structure model	
No. of reflections (Fo > 0σ(Fo))	30,525
Working set	28,955
Test set	1570
R_work_/R_free_ factor	0.21/0.25
Total atoms	9851
Wilson B factor (Å^2^)	55.4
Average B factor (Å^2^)	56.1
RMS deviations	
Bond lengths (Å)	0.012
Bond angles (°)	1.4
Ramachandran plot (%)	
Most favored	85.8
Allowed	14.2
Disallowed	0.0

Nevertheless, the αβ and αγ heterodimers in the apo β-mutant HsIDH3 structure also exhibit some notable conformational differences from those in the isolated forms^36,39^. In particular, the αβ heterodimer assumes an open overall conformation similar to that of the isolated αγ heterodimer rather than the compact conformation of the isolated αβ heterodimer, rendering it suitable for allosteric activation and catalytic reaction (see discussion later). In addition, the N-terminal (residues 1–14) of the γ subunit is disordered in all of our previously determined αγ structures regardless of the presence or absence of ligands^36–38^; however, a large portion of the N-terminal region (residues 5–14) of the αγ heterodimer is well-defined in the heterooctamer, which plays an important role in the formation and function of the heterooctamer (see discussion later). It is also noteworthy that the C-terminal of the β subunit is located on the structure surface and involved in the crystal packing, but is not involved in the assembly of the heterodimer, the heterotetramer, and the heterooctamer (Fig. 1). This explains why the crystals of the β-mutant HsIDH3 diffracted X-rays better than those of wild-type HsIDH3, and the β-mutant HsIDH3 has comparable biochemical and enzymatic properties as wild-type HsIDH3.

### The heterodimer–heterodimer interface in the α_2_βγ heterotetramer

**Fig. 2:**
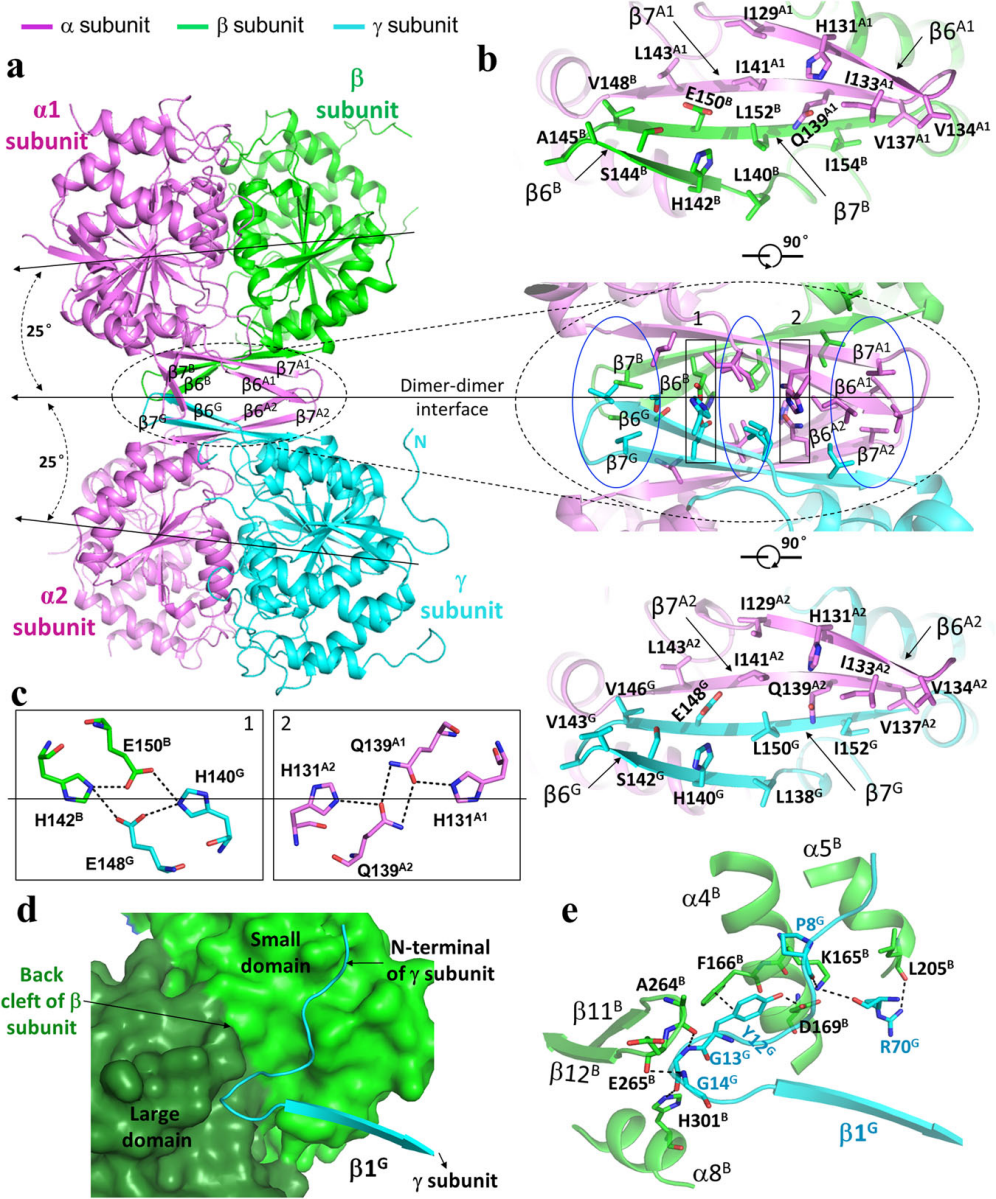
Interactions at the heterodimer–heterodimer and the heterotetramer–heterotetramer interfaces in the apo β-mutant HsIDH3 structure. **a** The α_2_βγ heterotetramer is assembled by the αβ and αγ heterodimers via their clasp domains. The color coding of the α, β, and γ subunits is the same as in Fig. 1. The pseudo 2-fold axis along the heterodimer–heterodimer interface, and the coplane axes of the αβ and αγ heterodimers are indicated. **b** Structure of the heterodimer–heterodimer interface. Middle panel: interactions at the interface consist of largely hydrophobic residues (marked by blue ovals) and a few hydrophilic residues (marked by black rectangles). Upper panel: interactions between the α and β subunits at the interface. Lower panel: interactions between the α and γ subunits at the interface. **c** Hydrogen-bonding interactions between the β and γ subunits (left panel) and between the two α subunits (right panel). **d** A surface diagram showing that the N-terminal region of the γ subunit (in cyan ribbon) of one heterotetramer lies in a shallow cleft (the “back cleft”) formed by the small and large domains of the β subunit (in green and dark green surface, respectively) of the other heterotetramer. **e** Interactions between the γ subunit of one heterotetramer (in cyan) and the β subunit of the other heterotetramer (in green). The hydrogen-bonding interactions are indicated with dashed lines.

In the structure of the β-mutant HsIDH3, the α_2_βγ heterotetramer is assembled by the αβ and αγ heterodimers via their clasp domains (Figs. 1 and 2a). There is a pseudo two-fold symmetry along the heterodimer–heterodimer interface, which is about 25° off the coplane axes of the αβ and αγ heterodimers. In other words, the coplane axes of the αβ and αγ heterodimers make a 50° angle. Thus, the heterotetramer has a distorted tetrahedron architecture with the two α subunits occupying two vertices on the same side and the β and γ subunits two vertices on the other side (Fig. 2a). The heterodimer–heterodimer interface buries about 804 Å^2^ solvent accessible surface or 3.0% of the surface area of each heterodimer. At the interface, the clasp β-sheets of the two heterodimers interact with each other to form a β-barrel in a reciprocal manner such that the clasp β-strands of the β subunit stack antiparallelly onto those of the α subunit of the αγ heterodimer, and the clasp β-strands of the γ subunit stack antiparallelly onto those of the α subunit of the αβ heterodimer (Fig. 2a, b). The interface consists of 22 hydrophobic residues and two Ser residues from the four clasp domains, which form extensive hydrophobic interactions (Fig. 2b). In addition, there are eight hydrophilic residues which form two layers of hydrophilic interactions to separate the hydrophobic interactions (Fig. 2b, c). Specifically, the side chains of His131^A1^ and Gln139^A1^ of the α subunit in the αβ heterodimer and His131^A2^ and Gln139^A2^ of the α subunit in the αγ heterodimer form one layer of hydrogen bonds, and the side chains of Glu150^B^ and His142^B^ of the β subunit and Glu148^G^ and His140^G^ of the γ subunit form another layer of hydrogen bonds (residues and structure elements of the α and β subunits of the αβ heterodimer and the α and γ subunits of the αγ heterodimer are superscripted by “A1” and “B”, and A2” and “G”, respectively). Sequence alignments showed that these residues are strictly or highly conserved in other mammalian NAD-IDHs and yeast NAD-IDH^35,39^ (Supplementary Fig. S5), suggesting that other eukaryotic NAD-IDHs might form the heterotetramers in a similar manner.

### The heterotetramer–heterotetramer interface in the (α_2_βγ)_2_ heterooctamer

In the structure of the β-mutant HsIDH3, the (α_2_βγ)_2_ heterooctamer is assembled by two α_2_βγ heterotetramers related by a crystallographic two-fold symmetry via the β and γ subunits (Fig. 1). Thus, the heterooctamer has a distorted tetrahedron architecture without a pseudo 222 symmetry. Specifically, the two β and two γ subunits are arranged alternately to form the inner core, and the four α subunits are positioned on the periphery. The heterotetramer–heterotetramer interface buries about 2248 Å^2^ solvent accessible surface or 4.3% of the surface area of the heterotetramer. At the interface, the N-terminal of the γ subunit of one heterotetramer intrudes into a shallow cleft between the small and large domains of the β subunit on the back of the pseudo allosteric site (“back cleft”) of the other heterotetramer (Fig. 2d). In particular, residues 10–14 (AKYGG) of the γ subunit make several hydrogen-bonding interactions with residues of the back cleft of the β subunit, which form a major part of the heterotetramer–heterotetramer interface (Fig. 2e). Specifically, the main chain of Pro8^G^ forms a hydrogen bond with the side chain of Lys165^B^; the side chain of Tyr12^G^ forms a hydrogen bond with the side chain of Asp169^B^ and a π–π stacking interaction with the side chain of Phe166^B^; the main chain of Gly13^G^ form a hydrogen bond each with the main chain of Ala264^B^ and the side chain of His301^B^; the main chain of Gly14^G^ forms a hydrogen bond with the main chain of Glu265^B^. In addition to the N-terminal, the α2 helix of the γ subunit also makes interactions with the α4 and α5 helices of the β subunit, which form a minor part of the heterotetramer–heterotetramer interface. In this region, the main chain and side chain of Arg70^G^ form a hydrogen bond each with the side chain of Lys165^B^ and the main chain of Leu205^B^, respectively. Sequence alignments showed that although the N-terminal of the γ subunit is different from that of the α and β subunits, residues 10–14 (AKYGG) are strictly or highly conserved in the regulatory subunits of other mammalian and yeast NAD-IDHs^35,39^ (Supplementary Fig. S5), suggesting that the N-terminal of the regulatory subunit in other eukaryotic NAD-IDHs might play a similar role in the assembly of the heterooctamer.

### The apo HsIDH3 structure assumes an inactive conformation

Our previous structural studies of the isolated αγ and αβ heterodimers show that the αγ heterodimer contains an allosteric site in the γ subunit to bind the activators CIT and ADP, and the binding of CIT and ADP induces conformational changes at the allosteric site which are transmitted to the active site via the heterodimer interface^36^. In the α^Mg^γ structure (PDB code: 5GRH), the side chain of Tyr135^G^ at the allosteric site assumes a conformation unsuitable for the CIT binding, the N-terminal region of the α7 helix at the heterodimer interface in both the α and γ subunits adopts a loop conformation, and the side chain of Tyr126^A2^ at the active site assumes a conformation unsuitable for the substrate ICT binding; and thus, the α^Mg^γ structure is considered to assume an inactive conformation^36^. On the other hand, in the α^Mg^γ^Mg+CIT+ADP^ structure (PDB code: 5GRF), the side chain of Tyr135^G^ at the allosteric site assumes a conformation to bind the CIT, the N-terminal region of the α7 helix in both the α and γ subunits adopts an α-helical conformation, and the side chain of Tyr126^A2^ at the active site assumes a conformation suitable for the ICT binding; and thus, the α^Mg^γ^Mg+CIT+ADP^ structure is considered to assume an active conformation^36^. In contrast, the αβ heterodimer contains a pseudo allosteric site in the β subunit, which is structurally different from the allosteric site and hence is unable to bind the activators^39^. In the α^Ca^β structure (PDB code: 6KDE), the N-terminal region of the α7 helix in both the α and β subunits assumes an α-helical conformation, and the key residues Tyr137^B^ at the pseudo allosteric site (equivalent to Tyr135^G^ at the allosteric site) and Tyr126^A1^ at the active site also assume the active conformations similar to those in the α^Mg^γ^Mg+CIT+ADP^ heterodimer; and therefore, the α^Ca^β structure is considered to assume an active conformation^39^. However, in the α^Ca^β structure, the αβ heterodimer assumes a more compact overall structure than the αγ heterodimer due to the rotation of the β subunit towards the α subunit, which leads to conformational changes of the heterodimer interface and consequently yields a distorted active site that cannot bind the metal ion appropriately in a catalysis relevant manner^39^. These results revealed the underlying molecular mechanisms of the isolated αγ and αβ heterodimers.

**Fig. 3:**
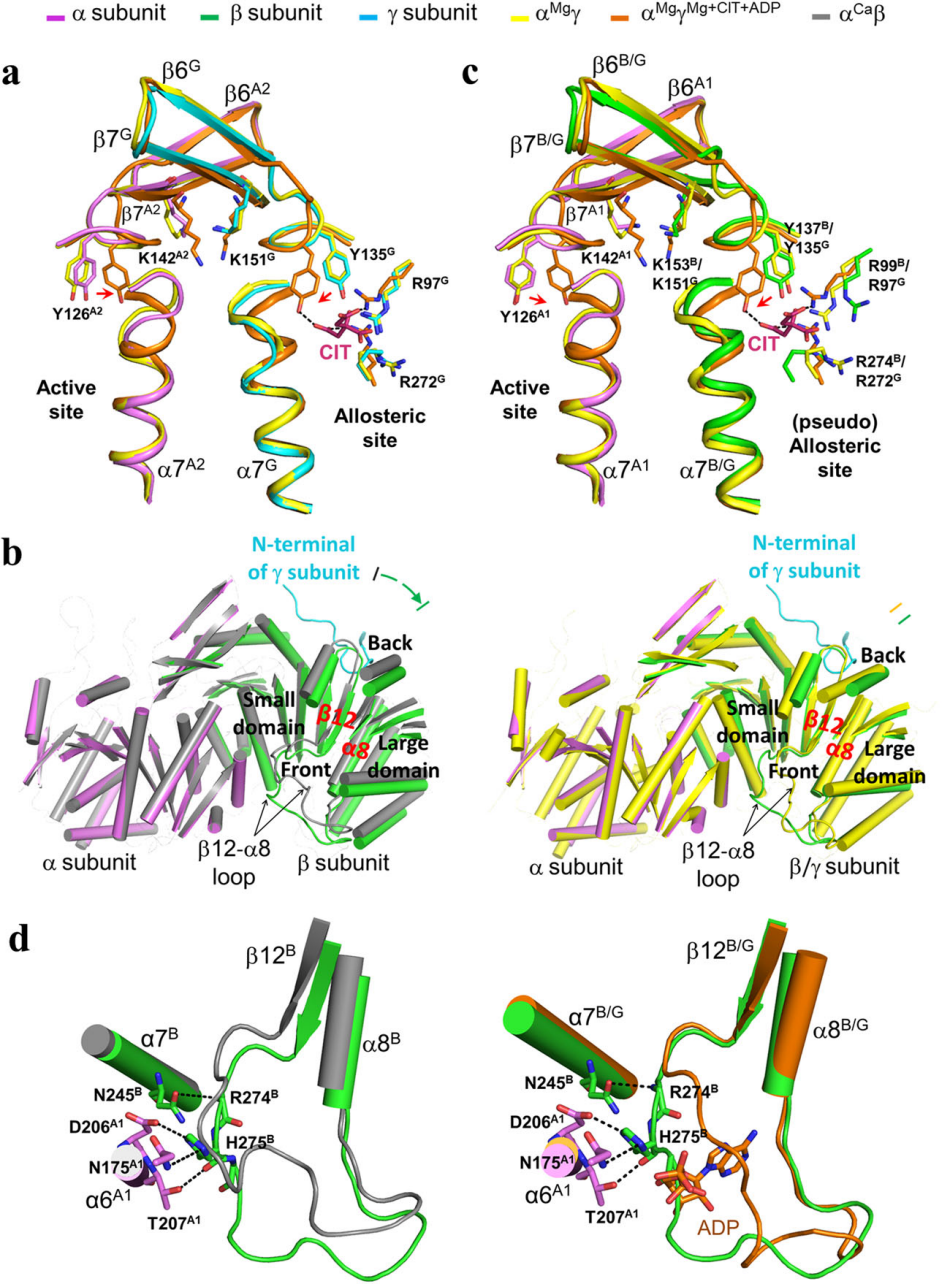
Structural comparisons of the αβ and αγ heterodimers in the apo β-mutant HsIDH3 and in the isolated forms. **a** Comparison of the αγ heterodimer in the heterooctamer of HsIDH3 and in the isolated forms at the heterodimer interface. The color coding of the subunits and structures is shown above. The key residues at the active site (Tyr126^A2^), the allosteric site (Arg97^G^, Tyr135^G^, and Arg272^G^), and the heterodimer interface (Lys151^G^ and Lys142^A2^) assume similar conformations as those in the inactive α^Mg^γ structure (PDB code: 5GRH) rather than those in the active α^Mg^γ^Mg+CIT+ADP^ structure (PDB code: 5GRF). **b** Comparison of the overall conformation of the αβ heterodimer in the heterooctamer of HsIDH3 with that of the isolated α^Ca^β heterodimer (PDB code: 6KDE) (in gray, left panel) and α^Mg^γ heterodimer (in yellow, right panel). The αβ heterodimer assumes an open overall conformation similar to that of the isolated α^Mg^γ heterodimer rather than the compact conformation of the isolated α^Ca^β heterodimer. For clarity, only the α helices and β strands are shown, and the loops are omitted except the β12-β8 loops of the β and γ subunits. The N-terminal of the γ subunit from another heterotetramer is also shown. **c** Comparison of the αβ heterodimer in the heterooctamer of HsIDH3 with the isolated α^Mg^γ heterodimer and α^Mg^γ^Mg+CIT+ADP^ heterodimer. The key residues at the active site (Tyr126^A1^), the pseudo allosteric site (Arg99^B^, Tyr137^B^, and Arg274^B^), and the heterodimer interface (Lys153^B^ and Lys142^A1^) assume similar conformations as those in the inactive α^Mg^γ structure rather than those in the active α^Mg^γ^Mg+CIT+ADP^ structure. **d** The β12^B^-β8^B^ loop of the β subunit in the heterooctamer of HsIDH3 exhibits some conformational differences from that in the isolated α^Ca^β heterodimer but still occupies the ADP-binding site in the α^Mg^γ^Mg+CIT+ADP^ structure. The hydrogen-bonding interactions of the β12^B^-β8^B^ loop with the α7^B^ and α6^A1^ helices are indicated with dashed lines.

Structural comparison shows that in the apo β-mutant HsIDH3 structure, the αγ heterodimer adopts an overall conformation similar to that in the α^Mg^γ structure with the inactive conformation rather than that in the α^Mg^γ^Mg+CIT+ADP^ structure with the active conformation^36^. In particular, the key residues at the active site (Tyr126^A2^) and the allosteric site (Tyr135^G^) assume inactive conformations, and the N-terminal regions of both α7^A2^ and α7 ^G^ helices at the heterodimer interface assume inactive loop conformations similar to those in the α^Mg^γ structure (Fig. 3a). Intriguingly, the αβ heterodimer exhibits some conformational differences from that in the α^Ca^β structure^39^. Structural analysis reveals that the insertion of the N-terminal of the γ subunit into the back cleft of the β subunit pushes the large domain of the β subunit to rotate away from the α subunit (the structure elements moving away from the α subunit by about 1.5–3 Å) (Fig. 3b). Consequently, the αβ heterodimer assumes an open overall conformation similar to that of the α^Mg^γ structure rather than the compact conformation of the α^Ca^β structure. In addition, the key residues at the active site (Tyr126^A1^) and the pseudo allosteric site (Tyr137^B^) also assume inactive conformations similar to those in the α^Mg^γ structure (Fig. 3c). The N-terminal region of helix α7^A1^ of the α subunit at the heterodimer interface assumes a loop conformation, but the N-terminal region of helix α7^B^ of the β subunit assumes an α-helical conformation. At the pseudo allosteric site, although the β3^B^–α3^B^ loop is disordered similar to that in the α^Ca^β structure, the β12^B^–α8^B^ loop exhibits some conformational differences from that in the α^Ca^β structure and in particular the N-terminal region of the β12^B^–α8^B^ loop maintains interactions with the α6^A1^ and α7^B^ helices at the heterodimer interface and still occupies the ADP-binding site, prohibiting the ADP binding (Fig. 3d). These results show that the formation of the heterooctamer renders the αβ heterodimer to adopt an overall conformation similar to that of the αγ heterodimer; however, the pseudo allosteric site remains incapable of binding the activators and thus the β subunit still has no regulatory function, explaining why the αβ heterodimer in the heterooctamer of HsIDH3 can be activated and has normal catalytic activity but cannot bind the activators. Taken together, our structural data indicate that the apo β-mutant HsIDH3 structure assumes an inactive conformation as both of the αβ heterodimer and the αγ heterodimer adopt inactive conformations similar to that in the α^Mg^γ structure.

### Functional roles of key residues in the assembly and allosteric regulation of HsIDH3

Our previous biochemical and structural studies of the αβ and αγ heterodimers showed that residues Arg97^G^, Tyr135^G^, and Arg272^G^ at the allosteric site, and residues Lys151^G^ and Lys142^A2^ at the heterodimer interface play important roles in the allosteric regulation^36^, whereas residues Arg99^B^, Tyr137^B^, and Arg274^B^ at the pseudo allosteric site play no notable role in the allosteric regulation^35,39^. Analysis of the apo β-mutant HsIDH3 structure also shows that residues His131^A1^, Gln139^A1^, His131^A2^, Gln139^A2^, His142^B^, Glu150^B^, His140^G^, and Glu148^G^ of the clasp domains play an important role in the assembly of the α_2_βγ heterotetramer (Fig. 2c). To investigate the functional roles of these residues in the allosteric regulation of HsIDH3, we prepared a series of HsIDH3 mutants containing point mutations of the key residues at the allosteric site (γ_R97A_, γ_Y135A_ and γ_R272A_) (Fig. 3a), the pseudo allosteric site (β_R99A_, β_Y137A_ and β_R274A_) (Fig. 3c), the heterodimer interfaces (α1_K142A_, α2_K142A_, α1_K142A_α2_K142A_, β_K153A_, and γ_K151A_) (Fig. 3a,c), and the heterodimer–heterodimer interface (α1_Q139A_, α2_Q139A_, α1_Q139A_α2_Q139A_, β_E150A_, and γ_E148A_) (Fig. 2c), and measured their kinetic parameters in the absence or presence of CIT and ADP to examine the effects of the mutations on the activity and allosteric activation of HsIDH3. Most of the HsIDH3 mutants could be expressed and purified well, and exist as heterooctamer in solution with high purity and homogeneity as shown by SEC and SDS-PAGE analyses (Supplementary Fig. S1). The mutant αβ and αγ heterodimers containing mutations α1_H131A_, α2_H131A_, β_H142A_, and γ_H140A_ could not be expressed and thus the HsIDH3 mutants containing these mutations could not be obtained.

**Fig. 4:**
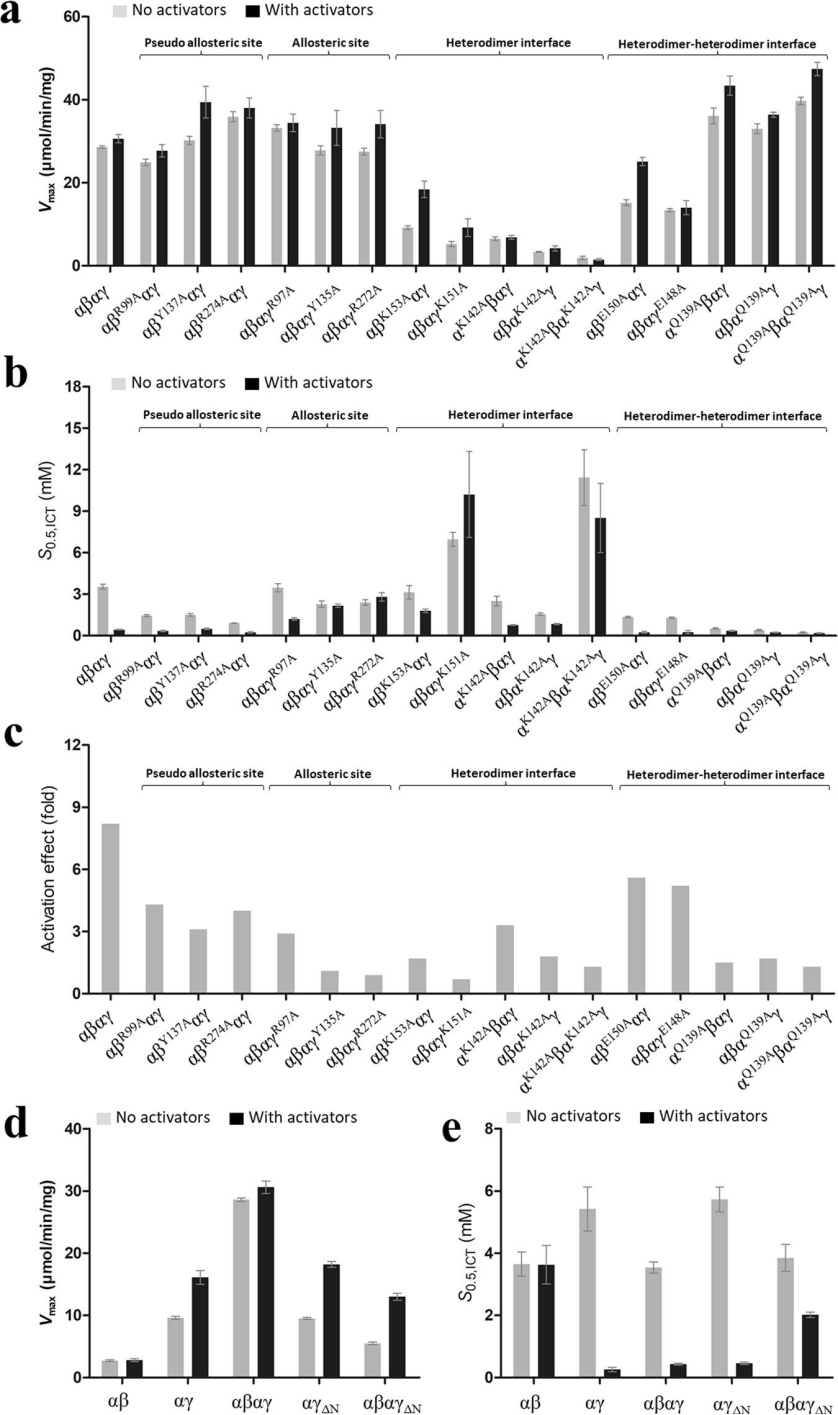
Effects of the mutations on the activity and allosteric activation of HsIDH3. **a** Graph presentations of the *V*_max_ values, **b** the *S*_0.5,ICT_ values, and **c** the activation effects of wild-type HsIDH3 and the HsIDH3 mutants containing mutations of key residues at the allosteric site, the pseudo allosteric site, the heterodimer interfaces, and the heterodimer–heterodimer interface in the absence or presence of CIT and ADP. The activation effect is defined as the ratio of the *S*_0.5,ICT_ in the absence and presence of the activators. The detailed kinetic parameters are listed in Table 2. **d** Graph presentations of the *V*_max_ values and **e** the *S*_0.5,ICT_ values of wild-type αβ and αγ heterodimers, wild-type α_2_βγ heterooctamer, the mutant αγ_ΔN_ heterodimer, and the mutant α_2_βγ_ΔN_ heterotetramer in the absence and presence of CIT and ADP. The detailed kinetic parameters are listed in Table 2.

Wild-type HsIDH3 exhibits a *V*_max_ of 28.6 μmol/min/mg and a *S*_*0.5*,ICT_ of 3.54 mM in the absence of the activators and a *V*_max_ of 30.6 μmol/min/mg and a *S*_*0.5*,ICT_ of 0.43 mM in the presence of the activators, displaying a significant activation effect (8.2 folds) (the ratio of the *S*_*0.5*,ICT_ in the presence and absence of the activators) (Table 2, Fig. 4 and Supplementary Fig. S3). Compared to wild-type HsIDH3, the HsIDH3 mutants containing mutations of the key residues at the allosteric site (γ_R97A_, γ_Y135A_, and γ_R272A_) exhibit comparable or slightly increased *V*_max_ (<1.2 folds) and comparable or slightly decreased *S*_*0.5*,ICT_ (<1.6 folds) in the absence of the activators, and exhibit comparable or slightly increased *V*_max_ (<1.1 folds) but significantly increased *S*_*0.5*,ICT_ (2.8–6.5 folds) in the presence of the activators, displaying no or weak activation effects (0.9–2.9 folds) (Table 2, Fig. 4 and Supplementary Fig. S3). These results indicate that the mutations at the allosteric site have significant impacts on the activation of HsIDH3. In contrast, the HsIDH3 mutants containing mutations of the key residues at the pseudo allosteric site (β_R99A_, β_Y137A_, and β_R274A_) exhibit comparable or slightly increased *V*_max_ (<1.3 folds) in both the absence and presence of the activators; however, the mutants exhibit moderately decreased *S*_*0.5*,ICT_ (2.4–3.9 folds) in the absence of the activators but comparable or slightly decreased *S*_*0.5*,ICT_ (0.9–1.9 folds) in the presence of the activators, displaying moderate activation effects (3.1–4.3 folds) (Table 2 and Fig. 4). These results indicate that the mutations at the pseudo allosteric site lead to partial activation of HsIDH3 in the absence of the activators but have no significant impacts on the allosteric regulation and thus the function of HsIDH3 in the presence of the activators. As the key residues at the allosteric site and the pseudo allosteric site are located in the heterodimer and heterodimer–heterodimer interfaces, it is possible that mutations of these residues might have some structural impacts on the heterodimer and heterodimer–heterodimer interfaces, which could contribute to the slightly increased activity and/or partial activation of the HsIDH3 mutants. Of note, the functional roles of Arg99^B^ and Arg97^G^ were examined by mutagenesis and biochemical studies previously, and the results showed that both residues are required for ADP activation^31^. This contradicts in part with our results, and the discrepancy could be due to the differed methods used in the preparations of the enzymes and the enzymatic activity assays.

**Table 2:** Activities and kinetic parameters of the wild-type and mutant HsIDH3^a^.

Enzyme	*V*_max_ (μmol/min/mg) −activators/+activators	*S*_0.5,ICT_ (mM) −activators/+activators	Activation effect (fold)^b^
αβ	2.72 ± 0.14/2.80 ± 0.23	3.65 ± 0.39/3.63 ± 0.62	1.0
αγ	9.62 ± 0.23/16.1 ± 1.1	5.42 ± 0.71/0.26 ± 0.07	20.8
α_2_βγ	28.6 ± 0.3/30.6 ± 1.0	3.54 ± 0.18/0.43 ± 0.03	8.2
*Pseudo allosteric site*			
M1: αβ_R99A_αγ	24.9 ± 0.8/27.7 ± 1.5	1.45 ± 0.07/0.34 ± 0.05	4.3
M2: αβ_Y137A_αγ	30.2 ± 1.0/39.4 ± 3.8	1.50 ± 0.09 /0.49 ± 0.05	3.1
M3: αβ_R274A_αγ	35.9 ± 1.2/38.0 ± 2.4	0.91 ± 0.01/0.23 ± 0.06	4.0
*Allosteric site*			
M4: αβαγ_R97A_	33.2 ± 0.8/34.4 ± 2.1	3.46 ± 0.29/1.20 ± 0.09	2.9
M5: αβαγ_Y135A_	27.8 ± 1.1/33.2 ± 4.2	2.28 ± 0.23/2.16 ± 0.12	1.1
M6: αβαγ_R272A_	27.5 ± 0.8/34.1 ± 3.3	2.40 ± 0.20/2.80 ± 0.30	0.9
*Heterodimer interface*			
M7: αβ_K153A_αγ	9.21 ± 0.43/18.4 ± 2.0	3.13 ± 0.49/1.80 ± 0.13	1.7
M8: αβαγ_K151A_	5.24 ± 0.63/9.19 ± 2.12	6.96 ± 0.50/10.2 ± 3.1	0.7
M9: α_K142A_βαγ	6.53 ± 0.44/6.84 ± 0.45	2.50 ± 0.35/0.76 ± 0.03	3.3
M10: αβα_K142A_γ	3.33 ± 0.11/4.20 ± 0.61	1.55 ± 0.10/0.84 ± 0.04	1.8
M11: α_K142A_βα_K142A_γ	1.92 ±± 0.40/1.48 ± 0.22	11.42 ± 2.01/8.50 ± 2.50	1.3
*Heterodimer–heterodimer interface*			
M12: αβ_E150A_αγ	15.2 ± 0.7/25.1 ± 1.0	1.34 ± 0.05/0.24 ± 0.07	5.6
M13: αβαγ_E148A_	13.4 ± 0.4/14.0 ± 1.7	1.29 ± 0.05/0.25 ± 0.12	5.2
M14: α_Q139A_βαγ	36.1 ± 1.9/43.4 ± 2.3	0.52 ± 0.04/0.35 ± 0.04	1.5
M15: αβα_Q139A_γ	33.0 ± 1.2/36.4 ± 0.6	0.40 ± 0.05/0.23 ± 0.03	1.7
M16: α_Q139A_βα_Q139A_γ	39.7 ± 0.9/47.4 ± 1.6	0.24 ± 0.03/0.18 ± 0.03	1.3
*Deletion of the N-terminal of the γ subunit (ΔN)*			
M17: αγ_ΔN_	9.51 ± 0.16/18.2 ± 0.47	5.73 ± 0.40/0.46 ± 0.04	12.5
M18: αβαγ_ΔN_	5.50 ± 0.21/13.0 ± 0.56	3.85 ± 0.43/2.02 ± 0.09	1.9
*Substitution of the C-terminal of the β subunit (β*_*mut*_*)*			
α_2_β_mut_γ	27.7 ± 0.6/34.0 ± 1.5	2.82 ± 0.21/0.40 ± 0.08	7.1

^a^The enzymatic activity and kinetic data were measured at standard conditions with varied concentrations of ICT in the absence or presence of the activators (CIT and ADP).

^b^Activation effect = *S*_0.5,ICT_ (no activators)/*S*_0.5,ICT_ (+activators).

The HsIDH3 mutants containing mutations of the key residues at the heterodimer interfaces (α1_K142A_, α2_K142A_, α1_K142A_α2_K142A_, β_K153A_, and γ_K151A_) exhibit moderately to significantly decreased *V*_max_ in both the absence (3.1–15.1 folds) and presence (1.7–20.7 folds) of the activators (Table 2, Fig. 4 and Supplementary Fig. S3). In addition, the mutants exhibit varied S_0.5_ (0.4–3.2 folds) in the absence of the activators, but moderately to significantly increased S_0.5_ (1.8–23.7 folds) in the presence of the activators, displaying no or moderate activation effects (0.7–3.3 folds). Although the α_K142A_βαγ mutant displays a moderate activation effect (3.3 folds), it exhibits a substantially decreased *V*_max_ (about 4.5 folds) in both the absence and presence of the activators. These results indicate that the mutations at the heterodimer interface significantly impair the communication from the allosteric site to the active sites of both α subunits and have severe impacts on the activation and function of HsIDH3.

For the key residues at the heterodimer–heterodimer interface, the HsIDH3 mutants containing mutations β_E150A_ and γ_E148A_ exhibit slightly decreased *V*_max_ (<2 folds) and slightly decreased S_0.5_ (<2.7 folds) in both the absence and presence of the activators, and display substantial activation effects (5.2–5.6 folds) (Table 2, Fig. 4 and Supplementary Fig. S3). These results indicate that the mutations have minor impacts on the activation and function of HsIDH3. Intriguingly, the HsIDH3 mutants containing mutations α1_Q139A_, α2_Q139A_, and α1_Q139A_α2_Q139A_ exhibit slightly higher *V*_max_ (about 1.2–1.6 folds) but significantly decreased *S*_0.5, ICT_ (6.8–14.8 folds) in the absence of the activators, and exhibit slightly higher *V*_max_ (about 1.2–1.5 folds) and slightly decreased *S*_0.5, ICT_ (1.2–2.3 folds) in the presence of the activators, displaying weak activation effects (<1.7 folds) (Table 2 and Fig. 4). These results indicate that the mutants are constitutively active regardless the absence or presence of the activators.

Taken together, our biochemical data demonstrate that the allosteric site plays a critical role and the pseudo allosteric site has no notable role in the allosteric regulation of HsIDH3; the heterodimer interfaces and the heterodimer–heterodimer interface play important roles in the allosteric regulation and function of HsIDH3.

### The N-terminal of the γ subunit is essential for the assembly and function of HsIDH3

In the apo β-mutant HsIDH3 structure, the N-terminal of the γ subunit of one heterotetramer inserts into the back cleft of the β subunit of the other heterotetramer to form the heterooctamer (Fig. 1). To validate the functional role of the N-terminal of the γ subunit in the assembly and function of HsIDH3, we removed the N-terminal region (residues 1–14) of the γ subunit (γ_ΔN_), and prepared the mutant αγ_ΔN_ heterodimer and α_2_βγ_ΔN_ heterotetramer. The mutant αγ_ΔN_ heterodimer and α_2_βγ_ΔN_ heterotetramer could be expressed and purified well with high purity and homogeneity as shown by SEC and SDS-PAGE analyses (Supplementary Fig. S1). SEC-MALS analyses show that like wild-type αγ heterodimer, the mutant αγ_ΔN_ heterodimer exists as a dimer with an average molecular weight of 84 kDa at low concentration (2 mg/ml) and a tetramer (presumably a dimer of heterodimers) with an average molecular weight of 123 kDa at high concentration (12 mg/ml) (Supplementary Fig. S2a and Table S1). However, unlike wild-type α_2_βγ which exists as a stable heterooctamer at both the low and high concentrations with an average molecular weight of about 284 kDa, the mutant α_2_βγ_ΔN_ heterotetramer exhibits an average molecular weight of 106 kDa which appears to be a mixture of the αβ and αγ_ΔN_ heterodimers and the α_2_βγ_ΔN_ heterotetramer at the low concentration, and an average molecular weight of about 125 kDa which appears to be a heterotetramer at the high concentration (Supplementary Fig. S2b and Table S1). These results indicate that the N-terminal deletion of the γ subunit does not affect the formation of the αγ heterodimer, but disrupts the formation of the heterooctamer, which are in agreement with the structural data showing that the N-terminal of the γ subunit is not involved in the formation of the αγ heterodimer but is involved in the formation of the heterooctamer. The biochemical data also suggest that the α_2_βγ_ΔN_ (possibly α_2_βγ) heterotetramer is unstable at low concentrations, and the formation of the heterooctamer stabilizes the formation of the α_2_βγ heterotetramer.

Consistently, the enzymatic activity assays show that the mutant αγ_ΔN_ heterodimer exhibits similar enzymatic properties as wild-type αγ heterodimer with comparable *V*_max_, *S*_0.5_, and activation effect (Fig. 4d and Table 2). However, compared to wild-type α_2_βγ, the mutant α_2_βγ_ΔN_ heterotetramer exhibits a significantly low activity in both the absence and presence of the activators and displays a weak activation effect (1.9 folds) (Fig. 4d and Table 2). Specifically, the mutant α_2_βγ_ΔN_ heterotetramer exhibits a *V*_max_ of 5.50 μmol/min/mg and a *S*_0.5_ of 3.85 mM in the absence of the activators, and a *V*_max_ of 13.0 μmol/min/mg and a *S*_0.5_ of 2.02 mM in the presence of the activators, which appear to be the averages of those of the αβ and αγ heterodimers. This is probably because at the standard assay conditions, the mutant α_2_βγ_ΔN_ heterotetramer has a very low concentration and exists mainly as a mixture of the αβ and αγ_ΔN_ heterodimers. Taken together, our biochemical data demonstrate that the N-terminal of the γ subunit plays an important role in the assembly and function of HsIDH3.

## Discussion

HsIDH3 exists and functions as a heterooctamer composed of the αβ and αγ heterodimers. Our previous biochemical studies showed that in HsIDH3, the α subunits in both αβ and αγ heterodimers have catalytic activity; the γ subunit plays a regulatory role, and the β subunit plays a structural role^35^. Our structural and biochemical studies of the αγ and αβ heterodimers revealed the underlying molecular mechanisms^36,39^. Specifically, the αγ heterodimer contains an allosteric site in the γ subunit and the binding of CIT and ADP to the allosteric site induces conformational changes of the active site via the heterodimer interface, leading to decrease of the *S*_0.5,ICT_ and hence activation of the enzyme^36^. In contrast, the αβ heterodimer contains a pseudo allosteric site in the β subunit, which is structurally different from the allosteric site and hence cannot bind the activators^39^. However, the structure, assembly and regulatory mechanism of HsIDH3 were unknown.

In this work, we determined the crystal structure of the apo HsIDH3 containing a β mutant. In the HsIDH3 structure, the αβ and αγ heterodimers form the α_2_βγ heterotetramer via their clasp domains, and two α_2_βγ heterotetramers assemble the (α_2_βγ)_2_ heterooctamer through the insertion of the N-terminal of the γ subunit of one heterotetramer into the back cleft of the β subunit of the other heterotetramer (Fig. 1). The functional roles of the key residues at the allosteric site, the pseudo allosteric site, the heterodimer interface, and the heterodimer–heterodimer interface, and the N-terminal of the γ subunit in the assembly and allosteric regulation of HsIDH3 are validated by mutagenesis and kinetic assays. The biochemical and structural data demonstrate that the α_2_βγ heterotetramer is unstable because the heterodimer–heterodimer interface is not very tight and involves mainly hydrophobic interactions, whereas the (α_2_βγ)_2_ heterooctamer is very stable because the two α_2_βγ heterotetramers interact with each other to form a ring-like architecture and the interfaces involve both hydrophilic and hydrophobic interactions (Figs. 1, 2, and Supplementary Fig. S2c, d). The formation of the (α_2_βγ)_2_ heterooctamer stabilizes the formation of the α_2_βγ heterotetramer. These findings reveal the molecular mechanism for the assembly of the heterotetramer and heterooctamer of HsIDH3.

Structural comparison shows that in the apo HsIDH3 structure, the αγ heterodimer assumes very similar overall conformation as the α^Mg^γ structure^36^, and the allosteric site assumes a proper conformation to bind the activators (Fig. 3a). However, the αβ heterodimer exhibits some conformational changes from the α^Ca^β structure^39^. The formation of the (α_2_βγ)_2_ heterooctamer renders the αβ heterodimer to adopt an open conformation similar to that of the α^Mg^γ structure rather than the compact conformation of the α^Ca^β structure (Fig. 3b). Nevertheless, the pseudo allosteric site remains unable to bind the activators (Fig. 3d). Hence, the α subunit of the αβ heterodimer in the heterooctamer of HsIDH3 can be allosterically activated and has normal catalytic activity but the β subunit still has no regulatory function. These results demonstrate that the structure characteristics and the regulatory mechanisms of the αβ and αγ heterodimers uncovered from the studies of the isolated αβ and αγ heterodimers are largely applicable to the heterooctamer of HsIDH3.

Our previous biochemical data showed that wild-type HsIDH3 exhibits a notably higher activity than the sum of the activities of the αβ and αγ heterodimers in both the absence and presence of activators, and the HsIDH3 mutant containing the α_Y126A_ mutation at the active site in either heterodimer exhibits about 50% of the activity of wild-type HsIDH3 and displays a significant activation effect; however, the HsIDH3 mutant containing the α_Y126A_ mutation in both heterodimers completely abolishes the activity^35^. These results indicate that in the heterooctamer of HsIDH3, both heterodimers have catalytic activity and can be activated by the activators, and the binding of the activators to the allosteric site in the γ subunit can regulate the α subunit in both heterodimers. The apo HsIDH3 structure shows that the allosteric site in the γ subunit could bind the activators but the pseudo allosteric site in the β subunit could not bind the activators, and that the overall conformation and the active-site conformation in both heterodimers are suitable for allosteric activation and catalytic function (Fig. 3). Consistently, the biochemical data show that the mutations at the allosteric site have significant impacts and the mutations at the pseudo allosteric site have insignificant impacts on the activation and function of HsIDH3, indicating that the allosteric site plays a critical role and the pseudo allosteric site plays no notable role in the allosteric regulation of HsIDH3 (Fig. 4 and Table 2). Moreover, the mutations at the heterodimer interfaces have severe impacts on the activation and function of HsIDH3, indicating that the heterodimer interfaces play a vital role in the communication from the allosteric site to the active sites of both α subunits (Fig. 4 and Table 2). Furthermore, while the β_E150A_ and γ_E148A_ mutations at the heterodimer–heterodimer interface have minor impacts on the activation and function of HsIDH3; the α_Q139A_ mutation in either or both heterodimers renders the HsIDH3 mutants constitutively active, indicating that the heterodimer–heterodimer interface plays an important role in the assembly and allosteric regulation of the α_2_βγ heterotetramer and the (α_2_βγ)_2_ heterooctamer (Fig. 4 and Table 2). Taken together, our data suggest that upon the binding of the activators to the allosteric site, the activation signal is transmitted from the allosteric site to the active sites in both αβ and αγ heterodimers through the heterodimer and heterodimer–heterodimer interfaces, leading to the activation of both heterodimers in the (α_2_βγ)_2_ heterooctamer. These findings reveal the molecular mechanism for the allosteric regulation of HsIDH3.

All eukaryotes contain NAD-IDHs to catalyze the decarboxylation of isocitrate in the TCA cycle. However, the composition of NAD-IDHs differs from low eukaryotes to high eukaryotes. In low eukaryotes such as *S. cerevisiae*, the NAD-IDH is composed of two types of subunits (ScIDH1 and ScIDH2) in 1:1 ratio, which form the ScIDH1/ScIDH2 heterodimer that further assembles the heterotetramer and the heterooctamer; ScIDH2 is the catalytic subunit and ScIDH1 is the regulatory subunit^19,40,41^. The yeast NAD-IDH structure exhibits a distorted tetrahedron architecture, in which the ScIDH1 subunits form the inner core and the ScIDH2 subunits are positioned on the outside surface, and thus the four ScIDH1 subunits are in two different structural environments with different conformations^20^.

In high eukaryotes such as human, the NAD-IDH is composed of three types of subunits (α, β, and γ) in 2:1:1 ratio, which form the αβ and αγ heterodimers that further assemble the α_2_βγ heterotetramer and the (α_2_βγ)_2_ heterooctamer. Although early biochemical studies of mammalian NAD-IDHs showed that the α subunit is the catalytic subunit and the β and γ subunits are the regulatory subunits^30–34^, our biochemical and structural studies of HsIDH3 demonstrated that the α subunits in both αβ and αγ heterodimers have catalytic activity, the γ subunit plays the regulatory role, and the β subunit plays the structural role^35,39^. Intriguingly, the apo HsIDH3 structure also exhibits a distorted tetrahedron architecture, in which the two β subunits and two γ subunits are arranged alternately to form the inner core and the four α subunits are positioned on the outer surface, and the two β subunits are in different structural environments with different conformations from the two γ subunits (Fig. 1).

Structural comparison shows that yeast NAD-IDH and human NAD-IDH (HsIDH3) exhibit almost identical architecture and could be superimposed very well (Supplementary Fig. S6a). The ScIDH1/ScIDH2 heterodimer and the (ScIDH1/ScIDH2)_2_ heterotetramer of yeast NAD-IDH have very similar structural topologies as the αγ and αβ heterodimers and the α_2_βγ heterotetramer of HsIDH3, respectively. The heterodimer, the heterodimer–heterodimer, and the heterotetramer–heterotetramer interfaces in the two enzymes are also very similar (Supplementary Fig. S6b–d). In particular, the N-terminal of ScIDH1 of one heterotetramer also interacts with the back cleft of ScIDH1 of another heterotetramer and is involved in the formation of the heterooctamer^20^ (Supplementary Fig. S6c). A detailed structural comparison in our previous work also showed that the allosteric site, the active site, and the heterodimer interface in the apo and CIT + AMP-bound ScIDH1/ScIDH2 heterodimer assume very similar structures as those in the α^Mg^γ and α^Mg^γ^Mg+CIT+ADP^ structure, respectively, although there are some small structural differences^36^ (Supplementary Fig. S6d). Intriguingly, in the CIT-bound and CIT + AMP-bound yeast NAD-IDH structures, the activator(s) bind to all four ScIDH1 subunits, which is in disagreement with the biochemical data showing that there are only two binding sites for the activators in yeast NAD-IDH^19^. This discrepancy might be due to the presence of excess CIT and AMP in the crystallization solution^20,42,43^. These results also suggest that like HsIDH3, only two of the four ScIDH1 subunits in yeast NAD-IDH have allosteric regulatory function and the other two have no regulatory function.

Sequence alignment of human NAD-IDH with yeast and other eukaryotic NAD-IDHs shows that the key residues composing the active site, the allosteric site, the pseudo allosteric site, the heterodimer interfaces, and the heterodimer–heterodimer interface, especially those involved in the conformational changes upon the binding of the activators and the structural communication from the allosteric site to the active sites are strictly or highly conserved^35,39^ (Supplementary Fig. S5). This suggests that all eukaryotic NAD-IDHs would assume a similar architecture and employ a similar allosteric regulation mechanism as human NAD-IDH.

## Materials and methods

### Cloning, expression, and purification

Wild-type αβ and αγ heterodimers and (α_2_βγ)_2_ heterooctamer of HsIDH3 were prepared as described previously^35^. The β-mutant HsIDH3 was also prepared as described previously^39^. Briefly, the DNA fragments encoding the α, β, and γ subunits of HsIDH3 were cloned into the co-expression vector pQlinkN with the C-terminals of the β and γ subunits attached with a TEV protease cleavage site and a His_6_ tag, yielding the pQlinkN-α-β-tev-His_6_ and pQlinkN-α-γ-tev-His_6_ plasmids. The plasmids were transformed into *E. coli* BL21 (DE3) Codon-Plus strain (Novagen). When the culture of the transformed cells reached an OD_600_ of 0.5, the protein expression was induced by 0.4 mM IPTG for 20 h at 24 °C. The cells were harvested and then sonicated on ice in the lysis buffer (50 mM HEPES, pH 7.4, 200 mM NaCl, 0.2 mM MnCl_2_, 10% glycerol, and 7.2 mM β-ME) supplemented with 1 mM PMSF. The target proteins were purified by affinity chromatography using a Ni-NTA column (Qiagen) with the lysis buffer supplemented with 20 mM and 200 mM imidazole serving as the washing buffer and elution buffer, respectively. The elution fraction was dialyzed overnight against the lysis buffer supplemented with TEV protease to cleave the His_6_-tag of the target protein. The cleavage mixture was reloaded on a Ni-NTA column and washed with the lysis buffer supplemented with 10 mM imidazole. The flow-through fraction containing the target protein was further purified by gel filtration using a Superdex 200 10/60 GL column (GE Healthcare) equilibrated with the storage buffer (10 mM HEPES, pH 7.4, 200 mM NaCl, and 5 mM β-ME). The (α_2_βγ)_2_ heterooctamer of HsIDH3 was prepared by co-purifying the separately expressed αβ and αγ heterodimers using the same methods as for the αβ and αγ heterodimers. The purities of the proteins were analyzed by 12% SDS-PAGE with Coomassie blue staining. The mutant αβ and αγ heterodimers and (α_2_βγ)_2_ heterooctamer of HsIDH3 containing point mutations were constructed using the QuikChange^®^ Site-Directed Mutagenesis kit (Strategene). Expression and purification of the mutants were carried out using the same methods as for the wild-type proteins.

### SEC-MALS analysis

The purities and molecular weights of the proteins were analyzed by an analytical light scattering instrument (SEC-MALS) consisting of an Agilent 1260 Infinity Isocratic Liquid Chromatography System, a Wyatt Dawn Heleos II Multi-Angle Light Scattering Detector, and a Wyatt Optilab T-rEX Refractive Index Detector (Wyatt Technology). Analytical size exclusion chromatography was performed at 24 °C using a Superdex 200 10/300 GL column (GE Healthcare) equilibrated with a mobile phase containing 10 mM HEPES (pH 7.4), 200 mM NaCl, and 5 mM β-ME. Hundred microliter protein solution was injected into the column and eluted at a flow rate of 0.4 ml/min. The column effluent was monitored simultaneously with three detectors for UV absorption, light scattering and refractive index. The data were analyzed using the ASTRA software (Wyatt Technology) to determine the molecular mass of the protein^44^.

### Crystallization, diffraction data collection, and structure determination

Crystallization was performed using the hanging drop vapor diffusion method at 20 °C by mixing equal volume of protein solution (10 mg/ml) and reservoir solution. Crystals of the β-mutant HsIDH3 grew in drops containing the reservoir solution of 0.05 M NH_4_Cl, 0.05 Bis-Tris (pH 6.5), and 30% pentaerythritol ethoxylate. Crystals were cryoprotected using the reservoir solution supplemented with 25% ethylene glycol. Diffraction data were collected at 100 K at BL17U1 of Shanghai Synchrotron Radiation Facility and processed with HKL3000^45^. Statistics of the diffraction data are summarized in Table 1.

The structure of the β-mutant HsIDH3 was solved with the molecular replacement method implemented in program Phaser^46^ using the structures of the α^Mg^γ heterodimer (PDB code 5GRH) and the α^Ca^β heterodimer (PDB code 6KDE) of HsIDH3 as the search models. Structure refinement was carried out with program Phenix^47^ and REFMAC5^48^. Model building was performed with program Coot^49^. Stereochemistry and quality of the structure model were analyzed using programs in the CCP4 suite^50^. Structure figures were prepared using PyMol^51^. Statistics of the structure refinement and the final structure model are also summarized in Table 1.

### Enzymatic activity assay

The enzymatic activities of wild-type and mutant αβ and αγ heterodimers and (α_2_βγ)_2_ heterooctamer of HsIDH3 were determined using the method as described previously^35^. The standard reaction solution (1 ml) consisted of 2 ng/ml enzyme, 33 mM Tris-acetate (pH 7.4), 40 mM ICT, 2 mM Mn^2+^, and 3.2 mM NAD. The activity is defined as the μmoles of NADH produced per min per milligram of enzyme (μmol/min/mg). The kinetic data in the absence of the activators (CIT and ADP) were measured with varied concentrations of ICT (0–40 mM), Mn^2+^ (0–10 mM), or NAD (0–10 mM) to obtain the *V*_max_ and *S*_0.5_ for ICT, Mn^2+^, or NAD, respectively. The kinetic data in the presence of the activators were measured at the same conditions supplemented with 1 mM CIT and 1 mM ADP. The kinetic parameters were obtained by fitting the kinetic data into the non-Michaelis–Menten equation “*V* = *V*_max_*[S]^h/(*S*_0.5_^h + [S]^h)” using program Graphpad Prism (Graphpad Software), where “[S]” is the concentration of ICT, Mn^2+^, or NAD; “*V*_max_” is the maximal velocity; “*S*_0.5_” is the apparent *K*_m_ (the Michaelis constant, the concentration of substrate at half-maximal velocity 0.5*V_max_, which may approximate the binding constant); and “h” is the Hill coefficient whose value depends on the number of substrate-binding sites and the number and type of interactions between these binding sites. All experiments were performed twice and the values were the averages of the measurements with the standard errors.

## Supplementary information

Supplementary information, Tables and Figures

## Data Availability

The crystal structure of the apo β-mutant HsIDH3 has been deposited in the Protein Data Bank with accession code 7CE3. All remaining data are contained within the article.

## Supplementary information

**Supplementary Information** accompanies the paper at (10.1038/s41421-020-00220-7).
