# Supplementary material for: Structure and allosteric regulation of human NAD-dependent isocitrate dehydrogenase
Source: Cell Discov. 2020 Dec 22;6:94. doi: 10.1038/s41421-020-00220-7 (PMC7752914; doi:10.1038/s41421-020-00220-7)
Supplement: Supplementary file 1 — Supplementary information, Tables and Figures [file 41421_2020_220_MOESM1_ESM.pdf]

Supplementary Table S1. SEC-MALS analysis results of the mutant $\alpha\gamma_{\Delta N}$ heterodimer, the mutant $\alpha_2\beta\gamma_{\Delta N}$ heterotetramer, and the mutant $\alpha_2\beta_{mut}\gamma$ heterooctamer in comparison with wild-type αβ and αγ heterodimers and wild-type $\alpha_2\beta\gamma$ heterooctamer.

Enzyme	Apparent molecular weight (kDa)		Reference
	2 mg/ml	12 mg/ml	
αβ	79	126	[35]
αγ	79	130	[35]
$\alpha\gamma_{\Delta N}$	84	123	This work
$\alpha_2\beta\gamma_{\Delta N}$	106	125	This work
$\alpha_2\beta_{mut}\gamma$	297	281	This work
$\alpha_2\beta\gamma$	284	283	This work

Supplementary Table S2. Enzymatic activities and kinetic parameters of wild-type and β-mutant HsIDH3[a].

Enzyme	No activators				With activators (CIT+ADP)	
	$V_{max,ICT}$ (μmol/min/mg)	$S_{0.5,ICT}$ (mM)	$S_{0.5,Mn}$ (μM)	$S_{0.5,NAD}$ (μM)	$V_{max,ICT}$ (μmol/min/mg)	$S_{0.5,ICT}$ (mM)
Wild-type HsIDH3	28.6±0.3	3.54±0.18	77.6±8.0	242±42	30.6±1.0	0.43±0.03
β-mutant HsIDH3	27.7±0.6	2.82±0.21	63.8±6.8	182±56	34.0±1.5	0.40±0.08

[a] The enzymatic activities and kinetic parameters of wild-type and β-mutant HsIDH3 were measured at the standard conditions with varied concentrations of the substrate ICT, the metal ion Mn^{2+}, or the co-factor NAD.

Supplementary Fig. S1 SEC and SDS-PAGE analyses of wild-type and mutant HsIDH3. (a) SEC analyses (Superdex 200 10/300 GL column) of wild-type (Wt) HsIDH3 ($\alpha_2\beta\gamma$), the β-mutant (β-mut) HsIDH3 ($\alpha_2\beta_{mut}\gamma$), and the HsIDH3 mutants containing point mutations at the allosteric site, the pseudo allosteric site, the heterodimer interface, and the heterodimer-heterodimer interface, as well as the mutant $\alpha\gamma_{\Delta N}$ heterodimer and the mutant $\alpha\beta\alpha\gamma_{\Delta N}$ heterotetramer. The abbreviations of the mutants are the same as in Table 2. **(b)** SDS-PAGE (12%) analyses of wild-type and mutant HsIDH3. M: molecular mass markers. The upper band represents the β (39 kDa) and γ subunits (39 kDa), and the lower band represents the α subunit (37 kDa).

a

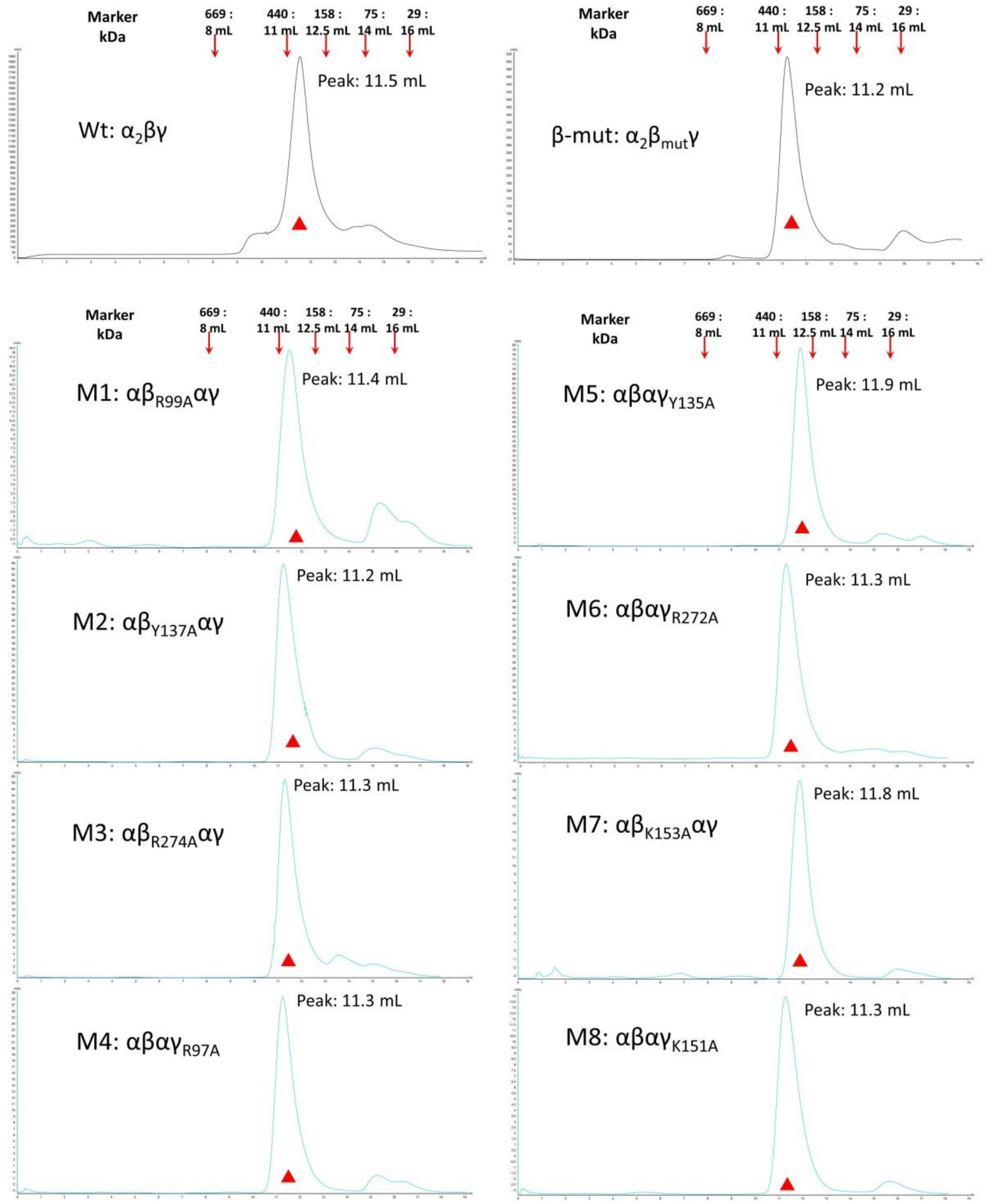

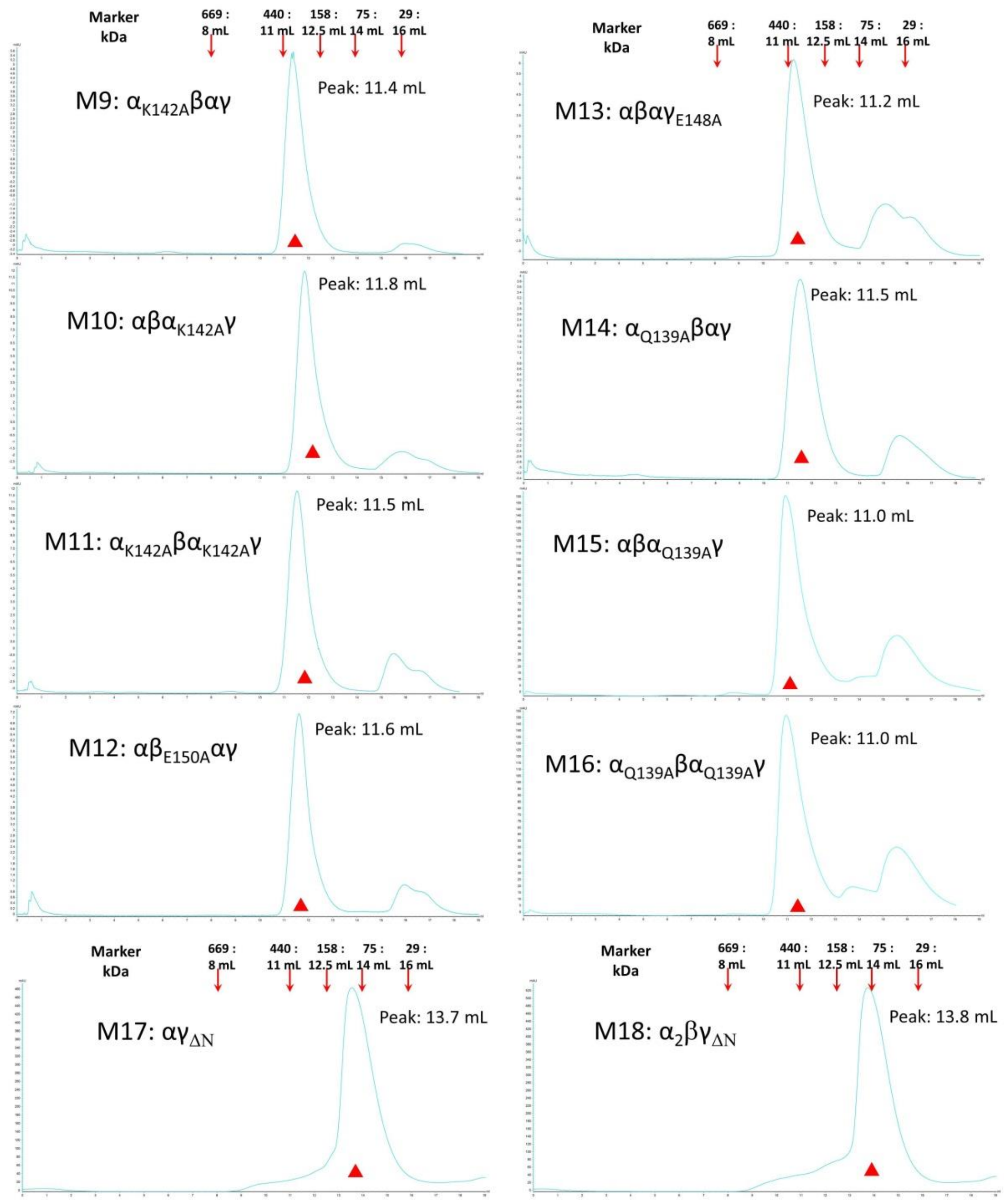
Marker kDa
669 : 8 mL
440 : 11 mL
158 : 12.5 mL
75 : 14 mL
29 : 16 mL
M9: $\alpha_{K142A}\beta\alpha\gamma$
Peak: 11.4 mL
M13: $\alpha\beta\alpha\gamma_{E148A}$
Peak: 11.2 mL
M10: $\alpha\beta\alpha_{K142A}\gamma$
Peak: 11.8 mL
M14: $\alpha_{Q139A}\beta\alpha\gamma$
Peak: 11.5 mL
M11: $\alpha_{K142A}\beta\alpha_{K142A}\gamma$
Peak: 11.5 mL
M15: $\alpha\beta\alpha_{Q139A}\gamma$
Peak: 11.0 mL
M12: $\alpha\beta_{E150A}\alpha\gamma$
Peak: 11.6 mL
M16: $\alpha_{Q139A}\beta\alpha_{Q139A}\gamma$
Peak: 11.0 mL
M17: $\alpha\gamma_{\Delta N}$
Peak: 13.7 mL
M18: $\alpha_2\beta\gamma_{\Delta N}$
Peak: 13.8 mL

b

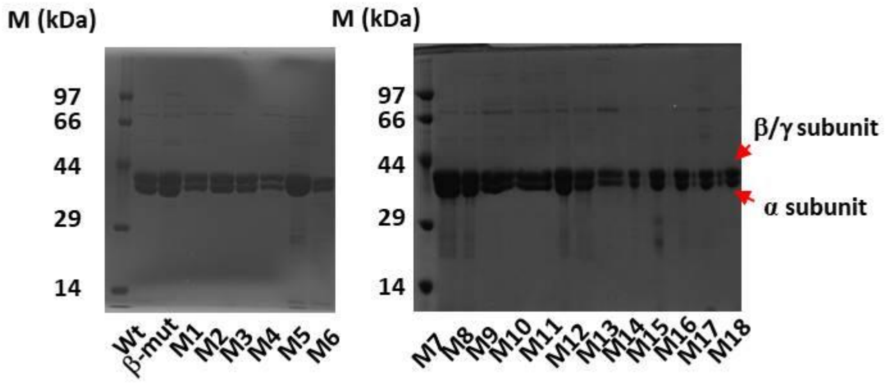
M (kDa)
97
66
44
29
14
Wt
β-mut
M1
M2
M3
M4
M5
M6
M7
M8
M9
M10
M11
M12
M13
M14
M15
M16
M17
M18
β/γ subunit
α subunit

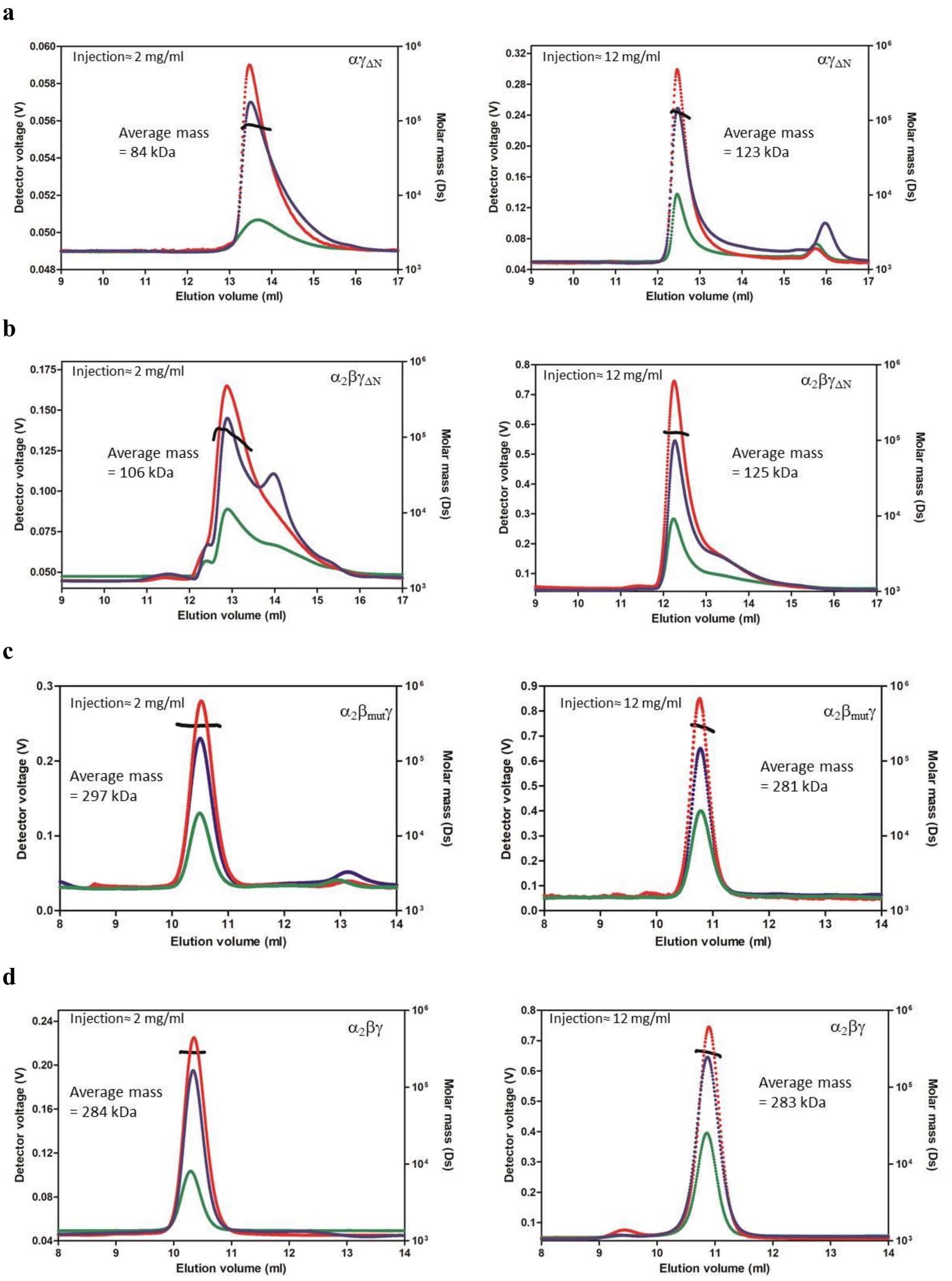

Supplementary Fig. S2 SEC-MALS analyses of the mutant $\alpha\gamma_{\Delta N}$ heterodimer, the mutant $\alpha_2\beta\gamma_{\Delta N}$ heterotetramer, and the mutant $\alpha_2\beta_{mut}\gamma$ heterooctamer of HsIDH3. **(a)** SEC-MALS analyses of the mutant $\alpha\gamma_{\Delta N}$ heterodimer at concentrations of 2 mg/ml and 12 mg/ml. **(b)** SEC-MALS analyses of the mutant $\alpha_2\beta\gamma_{\Delta N}$ heterotetramer at concentrations of 2 mg/ml and 12 mg/ml. **(c)** SEC-MALS analyses of the β-mutant HsIDH3 ($\alpha_2\beta_{mut}\gamma$) at concentrations of 2 mg/ml and 12 mg/ml. **(d)** SEC-MALS analyses of wild-type HsIDH3 ($\alpha_2\beta\gamma$) at concentrations of 2 mg/ml and 12 mg/ml. Chromatograms show the readings from the light scattering (red) at 90°, the refractive index (violet), and the UV (green) detectors. The left and right vertical axes represent the light scattering detector reading and the molecular mass. The black curve represents the calculated molecular mass.

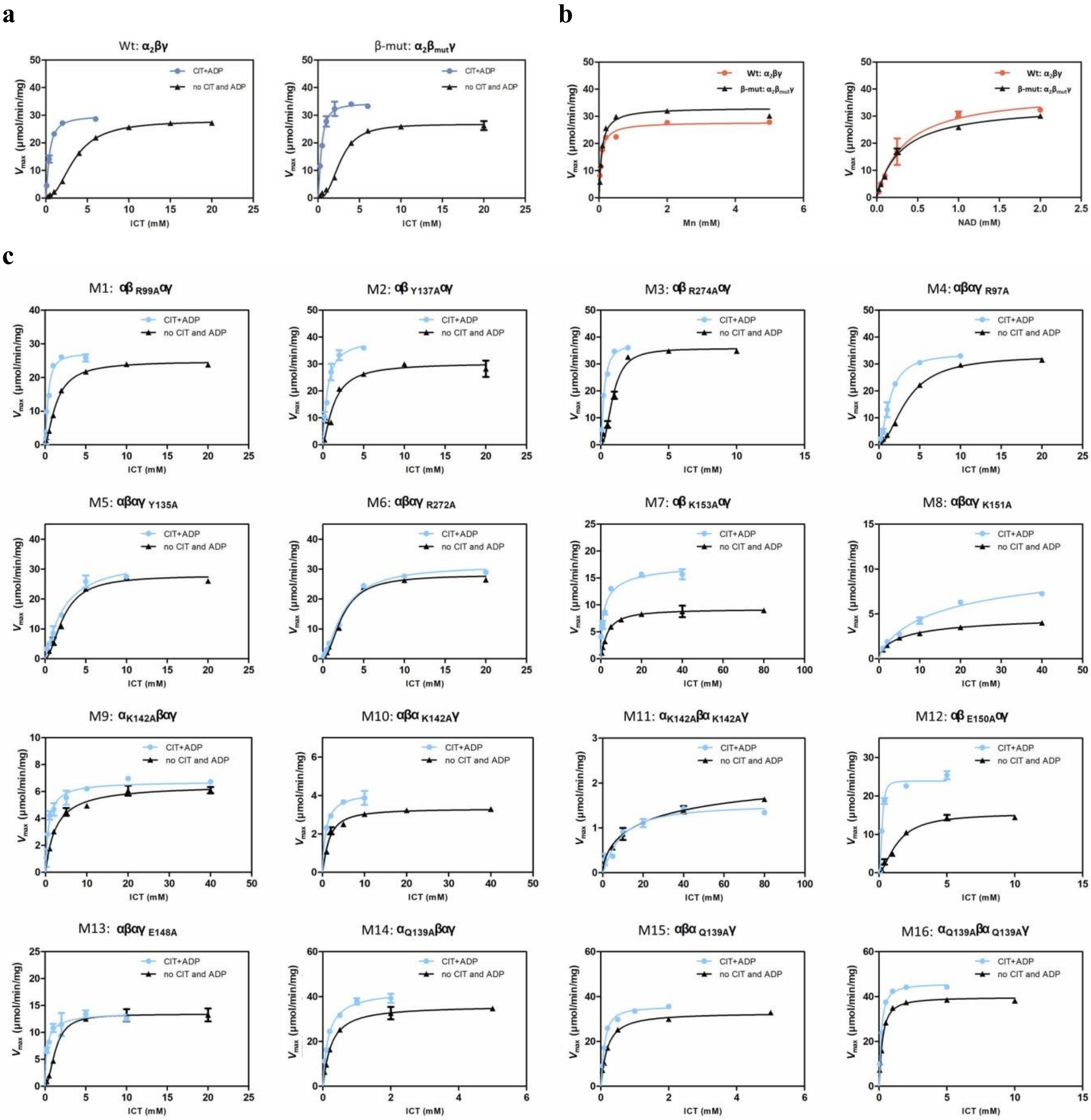

Supplementary Fig. S3 Saturation curves of wild-type and mutant HsIDH3. **(a)** ICT saturation curves of wild-type (Wt) HsIDH3 ($\alpha_2\beta\gamma$) (left panel) and the β-mutant (β-mut) HsIDH3 ($\alpha_2\beta_{mut}\gamma$) (right panel) in the absence and presence of the activators (CIT and ADP). **(b)** Mn^{2+} saturation curves of wild-type HsIDH3 and the β-mutant HsIDH3 (left panel) and NAD saturation curves of wild-type HsIDH3 and the β-mutant HsIDH3 (right panel) in the absence of the activators. **(c)** ICT saturation curves of the HsIDH3 mutants containing point mutations at the allosteric site, the pseudo allosteric site, the heterodimer interface, and the heterodimer-heterodimer interface in the absence and presence of the activators (CIT and ADP). The abbreviations of the mutants are the same as in Table 2. The activities were measured at the standard conditions with varied concentrations of the substrate ICT, the metal ion Mn^{2+}, or the cofactor NAD. The values are the averages of two independent measurements with the standard errors.

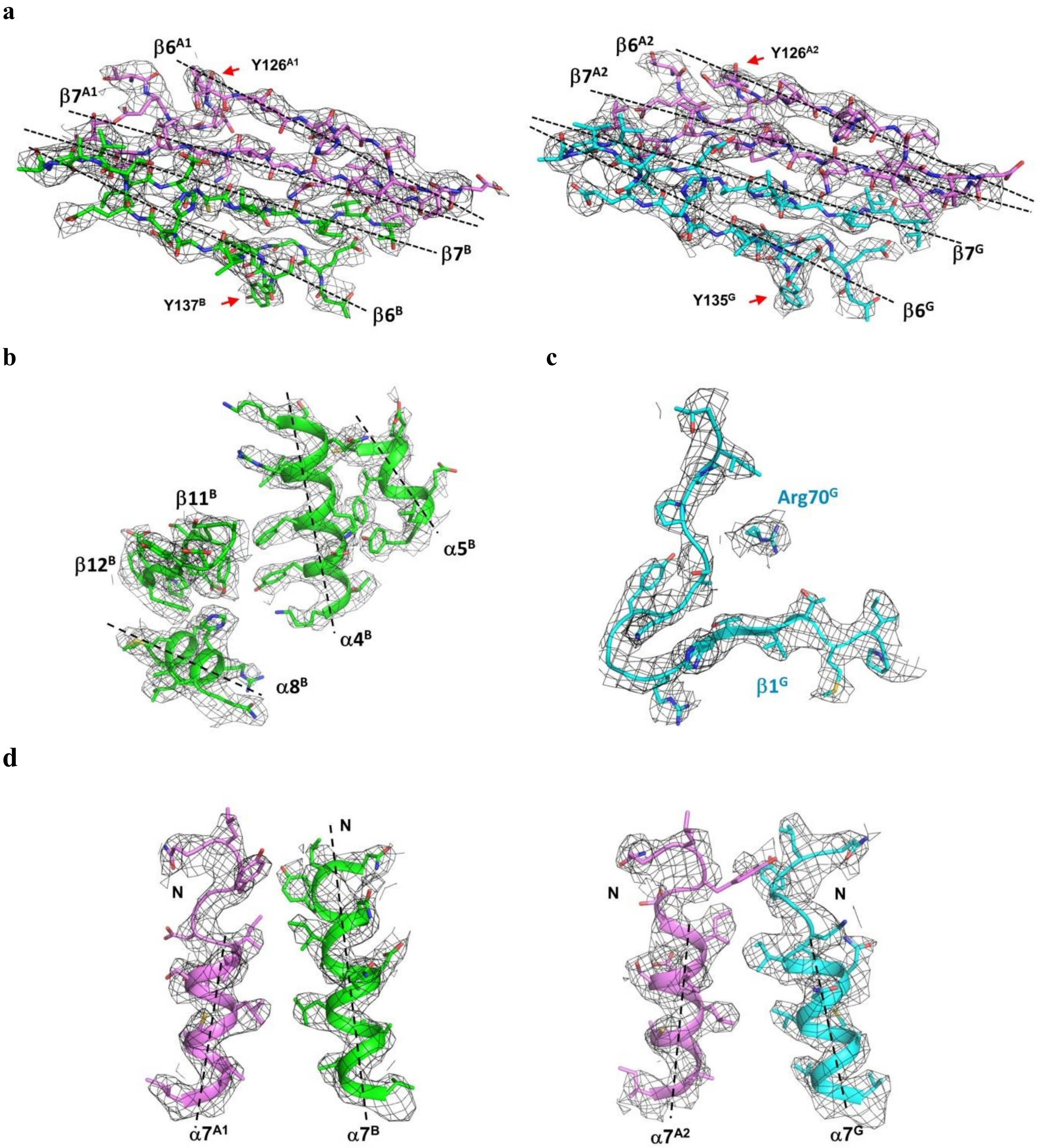

Supplementary Fig. S4 Simulated annealing composite omit maps (contoured at 1.0σ level) of several representative regions in the structure of the apo β-mutant HsIDH3. (a) The clasp domain of the αβ heterodimer (left) and the αγ heterodimer (right). **(b)** The back cleft of the β subunit. **(c)** The N-terminal of the γ subunit. **(d)** The N-terminal regions of the α7 helices of the αβ heterodimer (left) and the αγ heterodimer (right). Residues and structure elements of the α, β and γ subunits are colored in magenta, green and cyan, respectively.

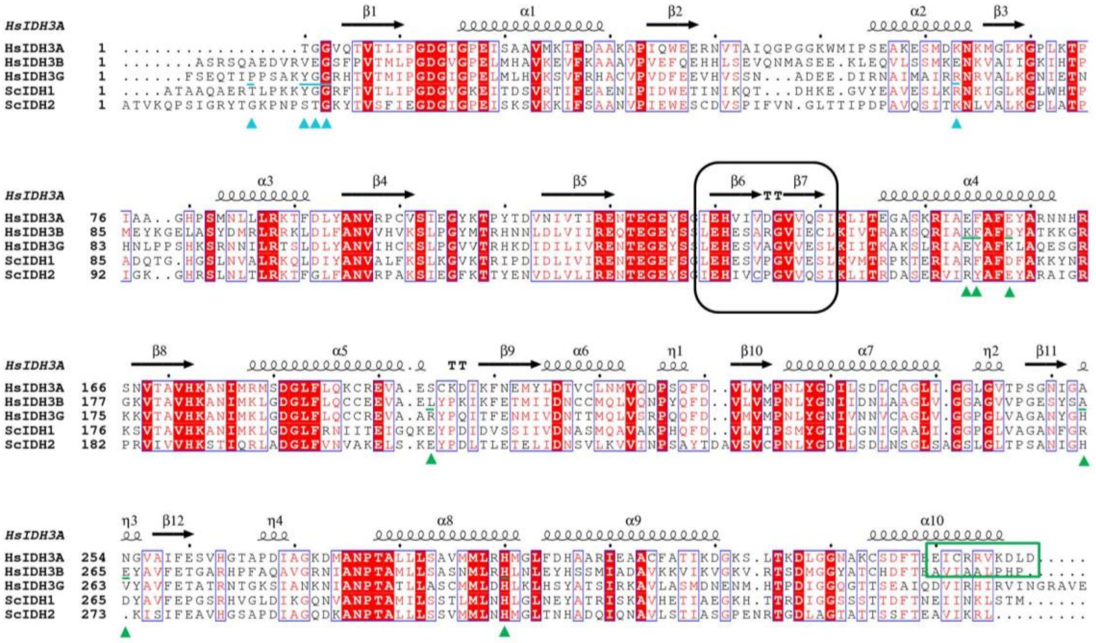

Supplementary Fig. S5 Sequence alignment of the α, β and γ subunits of human NAD-IDH (HsIDH3) and the ScIDH1 and ScIDH2 subunits of *S. cerevisiae* NAD-IDH. The β6 and β7 strands of the clasp domains which form the heterodimer-heterodimer interfaces are highlighted with black boxes. The residues of the γ subunit involved in the heterotetramer-heterotetramer interactions are indicated with cyan triangles, and the residues of the β subunit involved in the heterotetramer-heterotetramer interactions are indicated with green triangles. The C-terminal region of the β subunit that was substituted with the equivalent of the α subunit is highlighted with a green box.

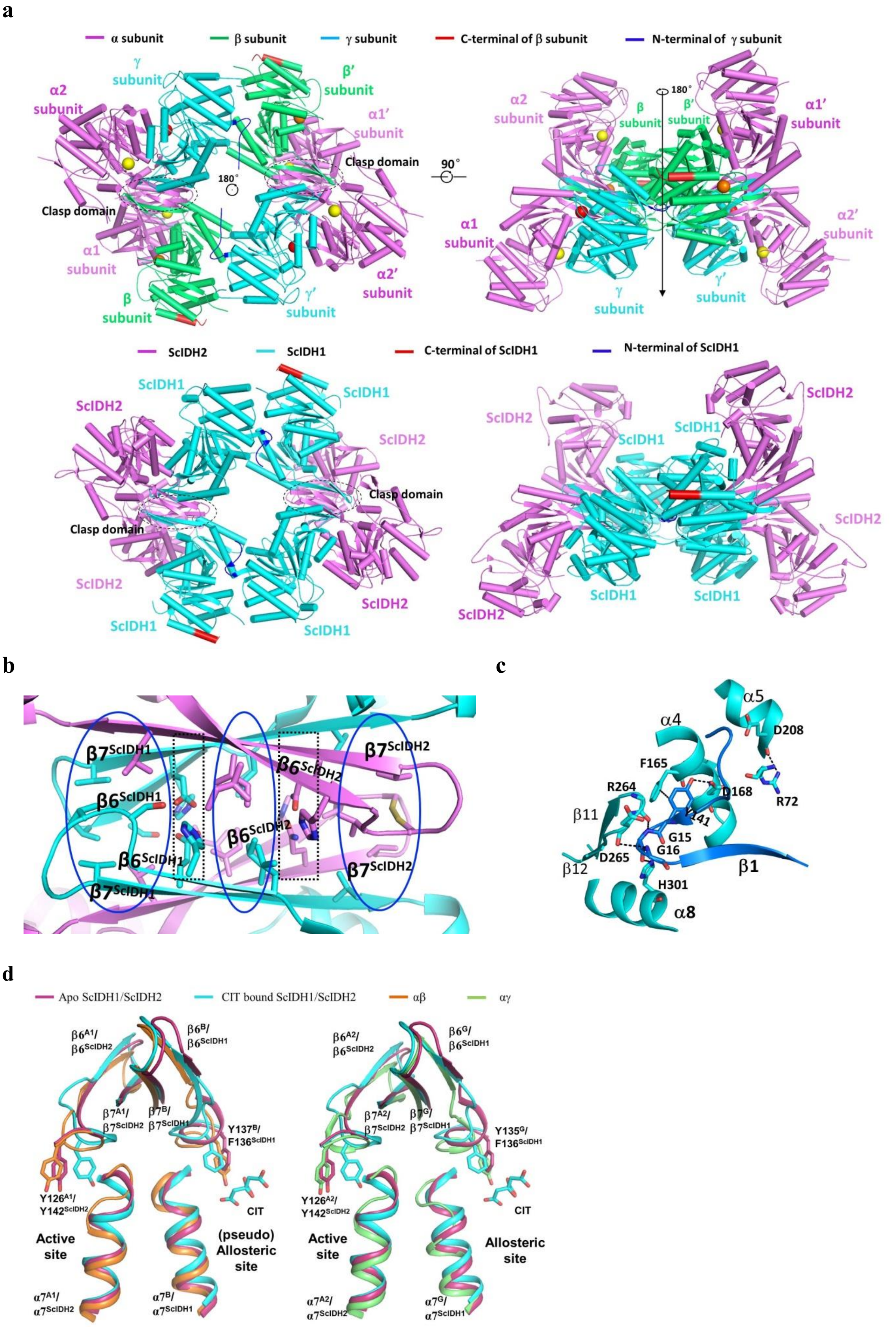
a
α subunit
β subunit
γ subunit
C-terminal of β subunit
N-terminal of γ subunit
γ subunit
β' subunit
α2 subunit
α1' subunit
Clasp domain
180°
90°
Clasp domain
α1 subunit
β subunit
γ' subunit
α2' subunit
α2 subunit
β subunit
β' subunit
α1' subunit
α1 subunit
α2' subunit
γ subunit
γ' subunit
ScIDH2
ScIDH1
C-terminal of ScIDH1
N-terminal of ScIDH1
Clasp domain
Clasp domain
b
c
α4
α5
D208
F165
R264
D168
R72
β11
Y141
G15
G16
D265
β12
β1
H301
α8
d
Apo ScIDH1/ScIDH2
CIT bound ScIDH1/ScIDH2
αβ
αγ
CIT
Active site
(pseudo) Allosteric site
Active site
Allosteric site

Supplementary Fig. S6 Structural comparison between human NAD-IDH (HsIDH3) and *S. cerevisiae* NAD-IDH. (a) Comparison of the overall structures of the apo β-mutant HsIDH3 (up panel) and the apo yeast NAD-IDH (PDB code: 3BLX) (low panel) in two different orientations. The color coding of the subunits is shown above. The N-terminal regions of the two γ subunits and the corresponding two ScIDH1 subunits are colored in blue, and the C-terminal regions of the two β subunits and the corresponding two ScIDH1 subunits are colored in red. The clasp domains of the αβ and αγ heterodimers and the ScIDH1/ScIDH2 heterodimers are indicated with dashed ovals. **(b)** Interactions at the heterodimer-heterodimer interface in the apo yeast NAD-IDH, showing a high similarity with those in the apo HsIDH3 (Fig. 2b). (**c)** Interactions between the N-terminal of one ScIDH1 subunit of one heterotetramer (in blue) and the back cleft of one ScIDH1 subunit of the other heterotetramer (in cyan) in the apo yeast NAD-IDH, showing a high similarity with those in the apo HsIDH3 (Fig. 2e). The hydrogen-bonding interactions are indicated with dashed lines. **(d)** Structural comparisons of the ScIDH1/ScIDH2 heterodimer in the apo and CIT-bound (PDB code: 3BLV) yeast NAD-IDH with the αβ heterodimer (left panel) and the αγ heterodimer (right panel) in the apo HsIDH3 at the heterodimer interfaces. The color coding of the subunits is shown above. The key residues at the active site (Tyr126 of the α subunit or Tyr142 of the ScIDH2 subunit) and the (pseudo) allosteric site (Tyr135 of the γ subunit, Tyr137 of the β subunit, and Phe136 of the ScIDH1 subunit) are shown with side chains.
